# Failure Load and Failure Modes of Indirect Posterior Restorations Combining Occlusal Coverage with Axial Extension: A Systematic Review and Exploratory Meta-Analysis of In Vitro Studies

**DOI:** 10.3390/jfb17100497

**Published:** 2026-10-01

**Authors:** Vlad Ștefan Proca, Andreea-Florentina Gavrilă, Alina-Mihaela Apostu, Adrian Doloca, Nicoleta Ioanid, Alice-Arina Pendefunda, Ovidiu Dumitru Aungurencei, Eugenia-Larisa Țarevici, Alice-Teodora Chiriac, Petronela Bodnar, Violina Budu, Raluca-Maria Vieriu, Vasilica Toma, Ioana Rudnic, Oana Țănculescu

**Affiliations:** Grigore T. Popa University of Medicine and Pharmacy Iasi, 700115 Iasi, Romania; vlad-stefan-proca@umfiasi.ro (V.Ș.P.); adrian.doloca@umfiasi.ro (A.D.); nicoleta.ioanid@umfiasi.ro (N.I.); alice.ciocan@umfiasi.ro (A.-A.P.); ovidiu.aungurencei@umfiasi.ro (O.D.A.); eugenia-tarevici@umfiasi.ro (E.-L.Ț.); alice-teodora.rotaru@umfiasi.ro (A.-T.C.); petronela_bodnar@umfiasi.ro (P.B.); violina_budu@umfiasi.ro (V.B.); raluca-maria.vieriu@umfiasi.ro (R.-M.V.); vasilica.toma@umfiasi.ro (V.T.); ioana.rudnic@umfiasi.ro (I.R.); oana.tanculescu@umfiasi.ro (O.Ț.)

**Keywords:** dental biomaterials, indirect restorations, vonlay, veneerlay, occlusal veneer, axial extension, fracture resistance, failure load, systematic review

## Abstract

Indirect posterior restorations combining complete occlusal coverage with extension onto an axial surface are described inconsistently, and their biomechanical advantage remains uncertain. This review compared failure load and a review-defined severe-failure category of morphology-defined axial-extension restorations with occlusal-only restorations and full-coverage crowns. PubMed, Scopus, Embase, and Web of Science Core Collection were searched from inception and last checked on 22 July 2026; citation searching supplemented the database searches. Eligible studies tested restorations meeting the morphological definition in extracted human posterior teeth. Two reviewers independently selected studies, extracted data, and appraised risk of bias using RoBDEMAT with QUIN as a supplementary appraisal. Multiple eligible intervention arms were combined, and shared comparators were counted once. Exploratory random-effects models estimated average effects across heterogeneous laboratory systems using ratios of means (RoMs) and mean differences (MDs) for failure load and risk ratios (RRs) for review-defined severe failure. Eight studies were included. Five independent studies contributed to the main comparison, restricted to single-surface axial-extension versus occlusal-only restorations. Failure-load estimates were inconclusive (RoM 0.98, 95% CI 0.88 to 1.09; MD −23 N, 95% CI −195 to 149). Severe-failure events were sparse and inconsistently defined across the source studies; exact counts were therefore primary, and the pooled RR was exploratory (RR 1.10, 95% CI 0.65 to 1.86). Three studies compared axial-extension restorations with crowns, but these heterogeneous comparisons were not pooled and remained inconclusive. Current in vitro evidence is insufficient to determine whether the evaluated axial-extension designs affect failure load or the occurrence of review-defined severe failure. Imprecision, methodological heterogeneity, and indirectness for clinical performance preclude claims of clinical superiority. A routine strength benefit of the evaluated axial-extension designs cannot be inferred. Standardized preparation definitions, testing protocols, and failure reporting are required. Registration: OSF, DOI 10.17605/OSF.IO/9PXE5 (retrospective).

## 1. Introduction

Tooth wear is the cumulative loss of dental hard tissue through mechanical and chemical processes. Its severity increases with age, but clinically relevant wear is also encountered in young adults [1,2], in whom early loss of occlusal anatomy may create a restorative problem despite the persistence of substantial sound enamel and dentin. When treatment is required, the central question is not only how to replace the missing tissue, but how to do so without unnecessarily sacrificing the tissue that remains.

This concern has driven a shift from retentive full-coverage preparations toward adhesively retained, defect-oriented restorations. In an experimental study of posterior preparation designs, complete-crown preparations removed approximately 67.5–75.6% of coronal tooth structure, whereas the least invasive adhesive and inlay designs removed approximately 5.5–27.2% [3]. Although these figures do not establish an indication for partial coverage in every case, they support conservative preparation when clinically feasible.

The biomimetic adhesive approach and additive rehabilitation protocols emphasize preservation of tooth structure and future treatment options [4,5,6,7]. Several indirect posterior designs have emerged within this conservative framework. Occlusal-only restorations—often called occlusal veneers or table-tops—replace lost occlusal tissue without a deliberate axial finish line. Other designs combine complete occlusal coverage with an extension onto a buccal, palatal, or lingual axial surface. In the laboratory literature eligible for this review, this morphology was described as a complete veneer in 2013 [8]; the term vonlay was subsequently used clinically for a restoration combining an onlay and a veneer [9].

Clinical cohorts have reported favorable survival for selected adhesively bonded lithium-disilicate posterior restorations, including partial-coverage designs [10,11,12], but these studies do not isolate the independent contribution of axial extension geometry and therefore cannot establish whether the axial extension itself improves or worsens mechanical performance or restoration survival.

The absence of standardized terminology is therefore more than an editorial problem. The same term has been applied to different preparations, and comparable preparations have been reported under different names. A search based only on vonlay or veneerlay would miss eligible experiments and could include morphologically ineligible ones. The present review accordingly defines the intervention by three operational eligibility criteria: complete occlusal coverage, extension beyond a cuspal slope onto at least one axial surface with an identifiable finish line, and extra-coronal fabrication before adhesive luting.

From a biomechanical perspective, axial-extension designs may differ from other partial-coverage posterior restorations, including occlusal-only restorations and conventional inlays and onlays, in the bonded surface area, preparation depth, finish-line position, remaining tooth structure, and load transfer. They may therefore influence both failure load and the structures involved in failure. These outcomes should be considered together because peak load alone does not indicate whether failure involves tooth or root structures. The available literature has reported conflicting findings and substantial variation in tooth types, materials, preparation depths, substrates, adhesive/luting protocols, ageing procedures, and loading geometries [13,14,15].

Previous systematic and scoping reviews have examined occlusal veneers more broadly [16,17,18], but we found none that applied the present morphology-based definition and jointly considered failure load and a review-defined severe-failure category comprising fractures extending below the cementoenamel junction or involving the root. The objectives of this review were therefore to identify and characterize the available in vitro evidence for restorations combining complete occlusal coverage with an extension onto at least one axial surface, synthesize their biomechanical outcomes in comparison with other posterior restorative designs or reference groups when sufficiently comparable data were available, assess the reliability and applicability of the evidence, and identify design and reporting variables requiring standardization in future experiments.

## 2. Materials and Methods

This systematic review was reported in accordance with PRISMA 2020 [19]. The completed PRISMA 2020 checklist is provided in the Supplementary Materials. The review was not prospectively registered, and no formal protocol was prepared; the methods of the completed review were retrospectively registered on the Open Science Framework (OSF; DOI: 10.17605/OSF.IO/9PXE5). Where meta-analysis was not appropriate, reporting followed the SWiM guideline [20].

### 2.1. Eligibility Criteria

Experimental in vitro studies were eligible if they used extracted natural human premolars or molars and evaluated an adhesively luted indirect or semi-direct restoration meeting the morphological definition below. At least one quantitative biomechanical outcome had to be reported for an eligible intervention arm: static failure load, fatigue or cyclic survival, cycles to failure, failure mode, or experimentally measured stress or strain.

An eligible intervention arm had to satisfy all three criteria: (1) complete coverage of the occlusal surface while preserving the cuspal morphology of the preparation; (2) an extension beyond a cuspal slope onto at least one buccal, palatal or lingual axial surface, terminating in an identifiable axial finish line; and (3) fabrication as a solid restorative unit before adhesive luting. The morphology-based criteria informed the search strategy and eligibility assessment; the terminology used by the study authors was not an eligibility criterion.

Studies were eligible irrespective of comparator type, provided that at least one experimental arm met the target morphological definition. Occlusal-only restorations, full-coverage crowns, sound teeth and other restorative designs were eligible as comparators or reference groups. Any arm independently satisfying the target morphological definition was classified as an eligible intervention arm.

Reports were excluded if they contained no eligible intervention arm or evaluated only artificial teeth or replicas, anterior teeth, direct restorations, preparations without complete occlusal coverage, preparations without an axial extension meeting the morphological definition above, flat occlusal preparations with complete removal of the cuspal anatomy, finite-element analysis alone, or outcomes unrelated to biomechanics.

### 2.2. Information Sources and Search Strategy

PubMed, Scopus, Embase, and Web of Science Core Collection were searched from inception without language or date restrictions. The initial searches were conducted during May–June 2026, and the broadened searches were conducted in June 2026. Because no controlled-vocabulary term captures the target geometry, the initial strategy combined author terms (including vonlay, veneerlay, complete veneer, and posterior full veneer) with morphology-based combinations of occlusal-coverage, axial-surface, and posterior-tooth terms. The first round retrieved 71 records (PubMed, *n* = 16; Scopus, *n* = 20; Embase, *n* = 18; Web of Science, *n* = 17). Based solely on the number of records retrieved, the review team agreed to broaden the search terms before any title/abstract screening, full-text assessment, or inspection of outcome data. The broadened second search round retrieved 458 records (PubMed, *n* = 92; Scopus, *n* = 147; Embase, *n* = 102; Web of Science Core Collection, *n* = 117). The results of both search rounds were imported into the same Rayyan review and deduplicated together before screening. Exact database-specific search strategies are provided in Supplementary Table S1. Backward and forward citation searching of included reports and relevant reviews was performed after database deduplication. All four databases were last checked on 22 July 2026; no record new to the review set was identified.

### 2.3. Study Selection and Data Extraction

Two reviewers independently screened the deduplicated records at the title/abstract and full-text stages in Rayyan, which concealed each reviewer’s eligibility decisions until both reviewers had completed the respective phase. Reviewers were not blinded to author names or publication dates; masking of journal titles was not documented. Disagreements were resolved by consensus, with a third reviewer available for adjudication if required. Cohen’s kappa was 0.90 at title/abstract screening and 1.00 at full-text assessment.

Two reviewers independently extracted data using a piloted form that captured tooth type and arch, intervention terminology and geometry, occlusal reduction, axial reduction or finish-line depth, restoration thickness, intracoronal-box depth and width, bonding substrate, material and fabrication method, surface conditioning, bonding and luting protocols, periodontal-ligament simulation, ageing, thermocycling, indenter geometry, loading direction, group size, any post-allocation exclusions and their reasons, mean failure load, standard deviation, and failure-mode counts. Discrepancies were resolved by consensus and, when required, adjudication by a third reviewer. Preparation dimensions were separated because tooth reduction, finish-line depth, and restoration thickness are related but not interchangeable variables. For each direct comparison, we assessed whether these dimensions were held constant between intervention and comparator arms. Values reported only graphically were independently estimated and cross-checked. Study authors were not contacted for missing or clarifying information. No unreported group-level event counts were imputed.

Failure modes were retained in the terminology of each primary report and harmonized only when the primary report permitted a defensible mapping. For comparative synthesis, review-defined severe failure comprised fracture extending below the cementoenamel junction or involving the root. This review-defined category was not treated as equivalent to a prospectively defined clinical judgment of non-restorability. Study-specific definitions and the review mapping are presented with the failure-mode results. A sensitivity analysis restricted to a common explicit restorability definition was not possible because the primary reports did not provide sufficiently consistent criteria. Sayed et al. reported failure-mode percentages only in aggregate; because events could not be assigned to individual groups, these data were excluded from comparative failure-mode synthesis [21].

Potential sample overlap was assessed by comparing authorship, laboratory methods, group descriptions, sample sizes, and descriptive statistics. Gierthmuehlen et al. reported that their control groups originated from the earlier experiment by Guess et al. and were tested using an identical setup [8,22]. To avoid double-counting the reused control data, Guess et al., as the original report, was retained in the quantitative synthesis. Because the full extent of the overlap could not be reconstructed reliably, Gierthmuehlen et al. was summarized narratively.

### 2.4. Risk of Bias and Confidence in the Evidence

Two reviewers independently applied RoBDEMAT as the primary domain-based instrument [23] and QUIN as a supplementary structured assessment [24], resolving disagreements by consensus and, when required, adjudication by a third reviewer. QUIN scores and corresponding risk-of-bias categories were retained for transparency but were not used as continuous measures of methodological quality or as the basis for synthesis-level confidence. The completeness and standardization of preparation-depth reporting were considered under reporting and specimen/preparation standardization; the choice of a particular depth was not itself classified as low or high risk of bias. Because no validated framework is universally accepted for grading confidence in this specific body of in vitro biomechanical evidence, no formal GRADE rating or overall categorical certainty label was assigned. Instead, each synthesis was interpreted with reference to the number of independent studies, risk-of-bias concerns, consistency, precision, and the applicability of the laboratory outcome to the inference being made. In vitro testing was considered directly relevant to the review’s biomechanical question, but indirect for claims about clinical survival, biological complications, or patient-important outcomes.

### 2.5. Synthesis and Statistical Analysis

The primary approach was a structured narrative synthesis grouped by comparator and outcome. Comparability was assessed with reference to tooth type, material, axial-extension geometry, preparation dimensions, adhesive/luting protocol, ageing, loading configuration, attrition, and risk of bias. Preparation depth was evaluated as a design and comparability variable by describing the reported occlusal reduction, axial reduction or finish-line depth, restoration thickness and intracoronal-box dimensions, and by determining whether these were matched within each direct comparison. Meta-analysis was considered only when at least three independent studies reported sufficiently compatible data; study count alone was not used to justify pooling. The primary quantitative comparison was restricted to five independent studies comparing single-surface axial-extension designs with occlusal-only restorations and was interpreted as an exploratory average across diverse laboratory systems, rather than as the effect of a standardized preparation. A broad analysis including all eligible axial-extension geometries was retained as a sensitivity analysis. Crown comparisons were not pooled because the three studies were clinically and technically heterogeneous. For failure load, ratio of means and mean difference in newtons were used as co-primary effect measures. The ratio of means provided a relative effect scale across the different baseline failure-load scales created by tooth type and laboratory protocol, whereas the mean difference retained the original measurement scale. Bias-corrected log ratios of means were calculated in metafor (measure = “ROM”, correct = TRUE) and back-transformed for presentation. Meta-regression or quantitative subgrouping by preparation depth was not undertaken because only five independent studies contributed to the primary comparison, several depths varied within studies, and depth was confounded with geometry, material, substrate, and ageing protocol.

To avoid unit-of-analysis errors, multiple eligible intervention arms within a study were combined using standard formulas for pooled means and standard deviations. Multiple independent comparator arms were combined in the same way. A shared comparator was entered only once. Consequently, each study contributed one independent effect estimate to a given meta-analysis. This approach sacrifices arm-level contrasts but avoids artificial precision from repeated use of the same controls.

Random-effects models used restricted maximum likelihood estimation of τ^2^ and the Hartung–Knapp adjustment for confidence intervals. Statistical heterogeneity was summarized by τ^2^, I^2^ and, when estimable, a 95% prediction interval. Exact counts and totals were the primary presentation of review-defined severe failure. For review-defined severe failure, all eligible axial-extension arms with group-resolved events were combined within study; this synthesis therefore included both single- and multi-surface geometries and was treated as exploratory. The pooled risk ratio used the same random-effects REML model with Hartung–Knapp adjustment; a 0.5 continuity correction was applied only to studies containing a zero cell. Because several event tables were sparse, an equal-effects Mantel–Haenszel risk-ratio analysis based directly on cell counts and without continuity correction in the pooled estimate was added as a sensitivity analysis. A leave-one-study-out analysis assessed the stability of the single-surface mean-difference estimate. The discrepant Guess value (833 N in Table I of the original article versus 883 N in the abstract) was evaluated in a separate mean-difference sensitivity analysis. Subgroup meta-analyses were not performed when fewer than three independent studies were available or when the proposed modifier was inseparable from study identity. Funnel plots and small-study-effect tests were not used because every synthesis included fewer than ten studies. Analyses were performed in R version 4.6.1 using metafor version 5.0-1 [25].

The operational failure-category harmonization, quantitative-analysis hierarchy, and sensitivity analyses were not prospectively specified.

## 3. Results

### 3.1. Study Selection

**Figure 1:**
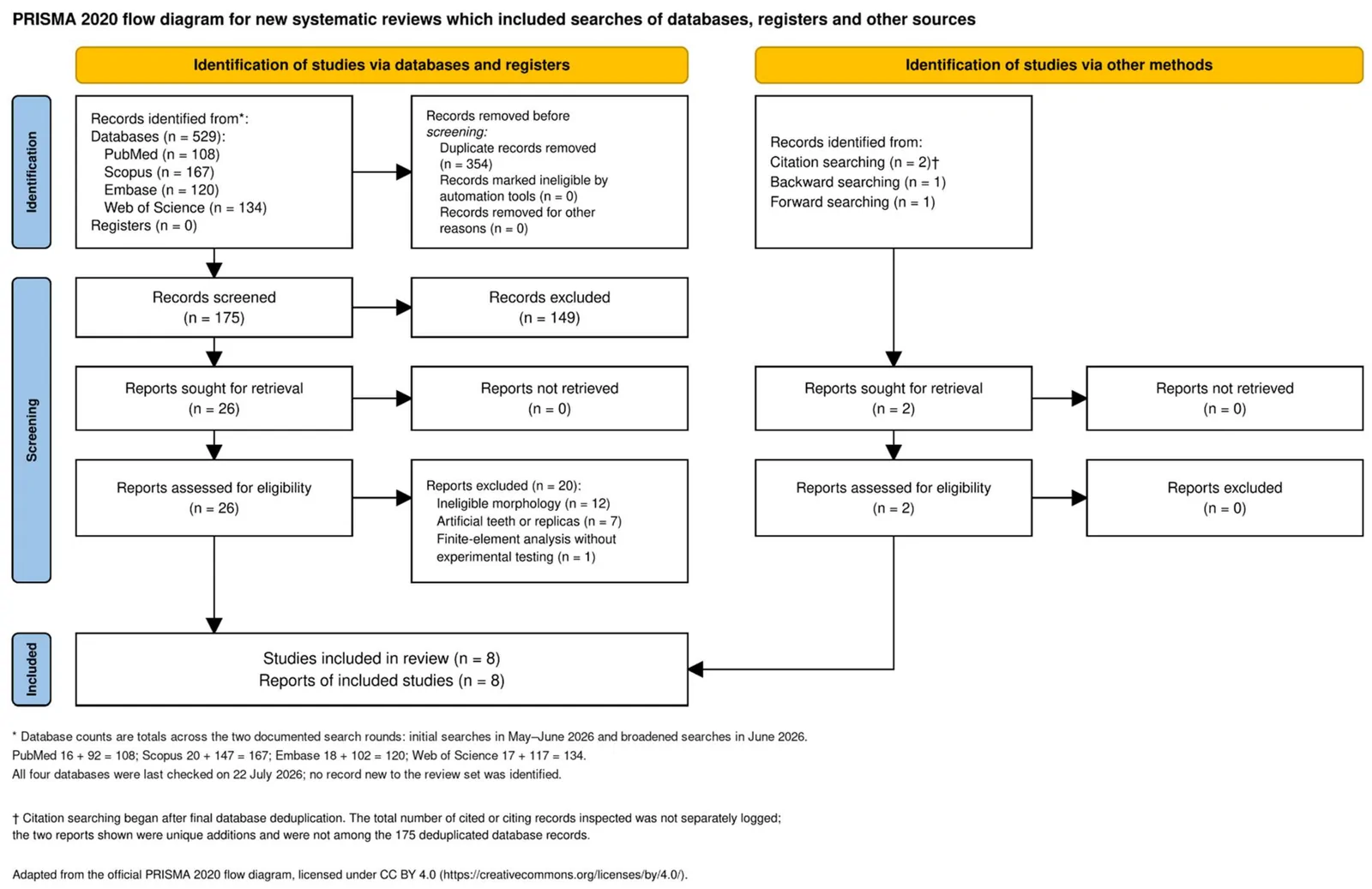
PRISMA 2020 flow diagram. Six reports included in the review were identified through database searching and two additional unique reports through citation searching: each report represented one study [19].

The two database-search rounds yielded 529 records. Deduplication of the combined database results in Rayyan removed 354 duplicate records, leaving 175 unique records for title/abstract screening. The database check on 22 July 2026 added no records to the review set. Of the 175 records, 149 were excluded, and 26 reports were retrieved and assessed for eligibility; six met the eligibility criteria, and 20 were excluded (Supplementary Table S3). Backward and forward citation searching identified two additional unique reports, both of which were retrieved, assessed, and included [21,26]. Thus, eight reports representing eight studies were included in the synthesis: six from database searching and two from citation searching [8,21,22,26,27,28,29,30] (Figure 1).

### 3.2. Study Characteristics

The eight studies were published between 2013 and 2026 [8,21,22,26,27,28,29,30]. Six used maxillary premolars, one used mandibular molars, and one used third molars without a clearly reported arch. Across eligible intervention arms, 264 specimens were allocated, and 263 were analysed. The one-specimen difference resulted from the exclusion of one specimen from the buccal-extension group in Moustafa et al. after apical fracture of the embedding epoxy resin. The same study excluded one specimen from the occlusal-only comparator for the same reason, leaving *n* = 7, *n* = 8, and *n* = 7 in the buccal-extension, lingual-extension, and occlusal-only groups, respectively [26]. In that report, the published failure-mode figure accounted for seven specimens in each group. No other clearly reconstructable post-allocation exclusions affected a quantitative comparison. Sayed et al. reported a 4% “abnormal fracture” exclusion that could not be reconciled with the reported integer group sizes or assigned to a specific group [21]. These data were therefore not included in the comparative failure-mode synthesis. The two fatigue cracks reported by Gierthmuehlen et al. were classified in this review as outcomes rather than withdrawals and were handled narratively [22].

Materials included pressed and CAD/CAM lithium disilicate, zirconia-reinforced lithium silicate, monolithic zirconia, polymer-infiltrated ceramic, 3D-printed nanohybrid composite, and semi-direct resin composite. Adhesive/luting protocols also varied across studies. Ageing ranged from seven-day storage without mechanical cycling to 1.2 million load cycles with thermocycling. Most static tests were applied approximately along the long axis, but Martins et al. applied the load at 135° to the long axis [28]; Moustafa et al. described vertical loading without clearly reporting its orientation relative to the tooth long axis [26], whereas Al-Mazzaly et al. reported the loading direction ambiguously [29]. Study characteristics are summarized in Table 1.

Preparation dimensions varied substantially. Reported occlusal reduction or intended occlusal restoration thickness ranged from 0.5 to 2.5 mm, while axial reductions or chamfer depths ranged from 0.3 to 1.0 mm. Intracoronal boxes, when present, were described using different combinations of depth, width, and cervical position. Importantly, the five studies contributing to the direct axial-extension versus occlusal-only comparison generally matched the occlusal reduction between the relevant intervention and comparator arms; therefore, their within-study contrasts were not simple comparisons of a thick versus a thin restoration. However, the magnitude and location of the axial reduction differed across studies, so the pooled estimates represent several preparation-depth protocols rather than the effect of one standardized depth. The reported dimensions and within-study comparability are detailed in Table 2.

### 3.3. Terminology and Morphology

Author terminology did not uniquely identify preparation geometry (Table 3). Designs meeting the review definition were labelled complete veneer, posterior minimally invasive full veneer, vonlay, veneerlay, occlusal veneer with buccal/lingual extension, and semi-direct occlusal veneer. Conversely, some reports using a vonlay-related term were excluded because the preparation did not provide complete occlusal coverage or did not extend onto an axial surface. This supports morphology-based eligibility and search strategies.

### 3.4. Risk of Bias

Methodological reporting was incomplete across the evidence base. Common limitations were no reported outcome-assessor blinding, incomplete reporting of randomization or allocation, small groups, limited justification of sample size, and incomplete standardization of natural-tooth morphology and verification of achieved preparation dimensions. Preparation depths were reported in all included studies, but the measurement reported was not uniform: some authors reported tooth reduction, others restoration thickness or finish-line depth, and box dimensions were not described in a common format. This limits cross-study comparability but does not make a selected depth intrinsically methodologically correct or incorrect. An a priori sample-size calculation was clearly reported by Al-Mazzaly et al. and Moustafa et al.; Martins et al. reported a power calculation, but its timing could not be confirmed as a priori [26,28,29]. Sayed et al. had the greatest reporting concerns and contained internal inconsistencies [21]. Industry support reported in Guess et al. and Gierthmuehlen et al. was treated separately as a funding characteristic and was not coded as methodological risk of bias [8,22]. Table 4 summarizes the reconciled assessments.

QUIN was used only as a supplementary structured assessment; its scores and categories should not be interpreted as overall measures of experimental validity or clinical applicability. Item-level judgments and supporting rationales are provided in Supplementary Table S2.

### 3.5. Failure Load: Axial Extension Design Versus Occlusal-Only Restoration

**Figure 2:**
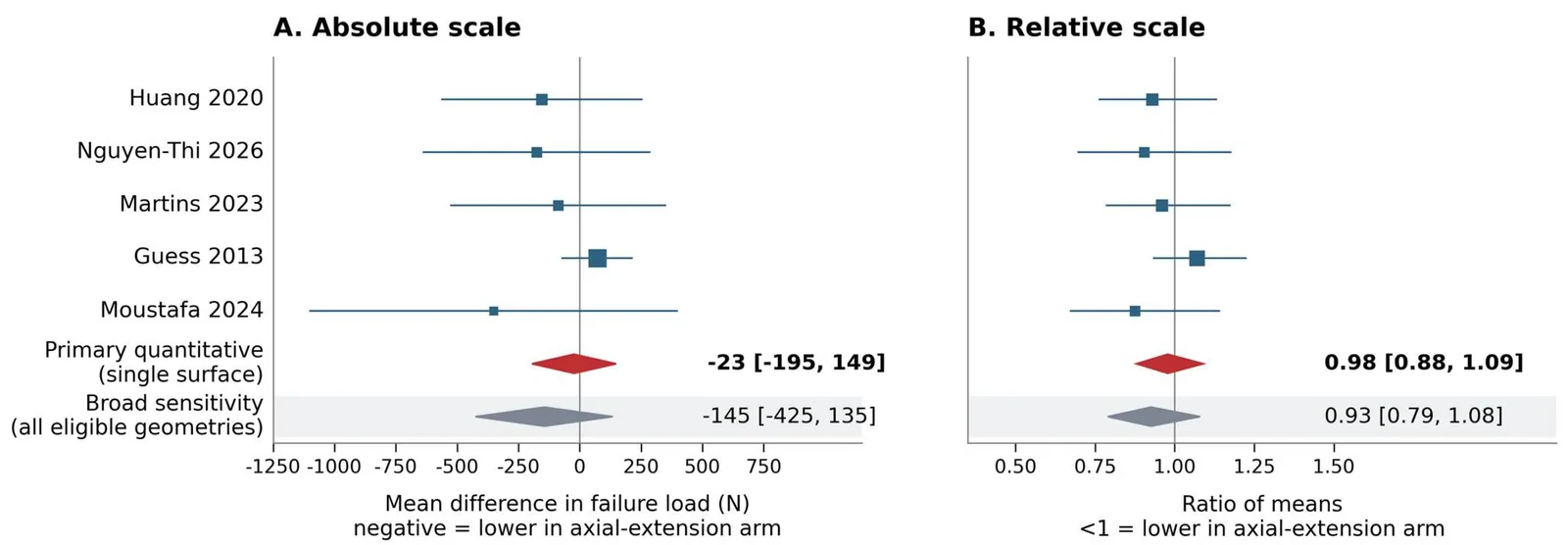
Failure-load estimates on absolute and relative scales for axial-extension versus occlusal-only restorations. Study rows and red diamonds represent the primary single-surface comparison; grey diamonds represent the broad all-eligible-geometry sensitivity analysis. Negative mean differences and ratios of means below 1 indicate lower failure load in the axial-extension arm. Independent strata were combined within studies, and shared comparators were counted once. Both summaries average distinct geometry–material–testing systems rather than one standardized preparation [8,26,27,28,30].

Five independent studies contributed to the primary quantitative comparison, which was restricted to single-surface axial-extension designs. After eligible intervention arms were combined within studies and shared comparators were counted once, the RoM was 0.98 (95% CI 0.88 to 1.09; I^2^ = 4.1%; 95% prediction interval 0.87 to 1.10), and the pooled MD was −23 N (95% CI −195 to 149; I^2^ = 13.7%; τ^2^ = 5718 N^2^; 95% prediction interval −294 to 248 N) (Figure 2). The estimates are compatible with both a relevant reduction and a relevant increase in failure load; they do not establish equivalence or absence of an effect.

The broad sensitivity analysis, which included all eligible geometries, produced a similar interpretation: the RoM was 0.93 (95% CI 0.79 to 1.08; I^2^ = 50.6%; 95% prediction interval 0.68 to 1.26), and the MD was −145 N (95% CI −425 to 135; I^2^ = 54.5%; τ^2^ = 34,826 N^2^; 95% prediction interval −734 to 444 N). Both analyses estimate average effects across heterogeneous combinations of geometry, material, and testing conditions rather than the effect of one standardized axial-extension design. Using the discrepant 883 N abstract value instead of the 833 N table value for Guess et al. produced a primary MD of −23 N (95% CI −209 to 163) and a broad-analysis MD of −141 N (95% CI −429 to 146); the interpretation was unchanged. Leave-one-study-out estimates ranged from +4 N after omission of Huang et al. to −161 N after omission of Guess et al. Only the estimate obtained after omission of Guess et al. had a 95% confidence interval that excluded zero (from −294 to −27 N), indicating that the pooled MD was sensitive to omission of this study. Study-level outcome data are provided in Supplementary Table S4.

Intracoronal-box configuration and functional-cusp status could not be evaluated as effect modifiers. The box-present evidence for the direct occlusal-only comparison came essentially from Guess et al., so the box effect was inseparable from study, material, and preparation protocol. The functional-cusp observation involved only Huang et al. and Moustafa et al. and was additionally confounded by arch and tooth type.

### 3.6. Failure Load: Axial Extension Design Versus Full-Coverage Crown

Three studies contributed independent comparisons with full-coverage crowns. Because the studies differed in materials, geometries, ageing procedures and loading protocols, and because their estimates pointed in opposite directions, they were not pooled. Huang et al. reported MD +283 N (95% CI +86 to +481), and Nguyen-Thi et al. MD +431 N (95% CI −6 to +867), numerically favouring the axial-extension designs, whereas Sayed et al. reported MD −270 N (95% CI −335 to −206), favouring the crown. These data do not support equivalence, superiority, or non-inferiority of either design.

### 3.7. Review-Defined Severe Failure

**Figure 3:**
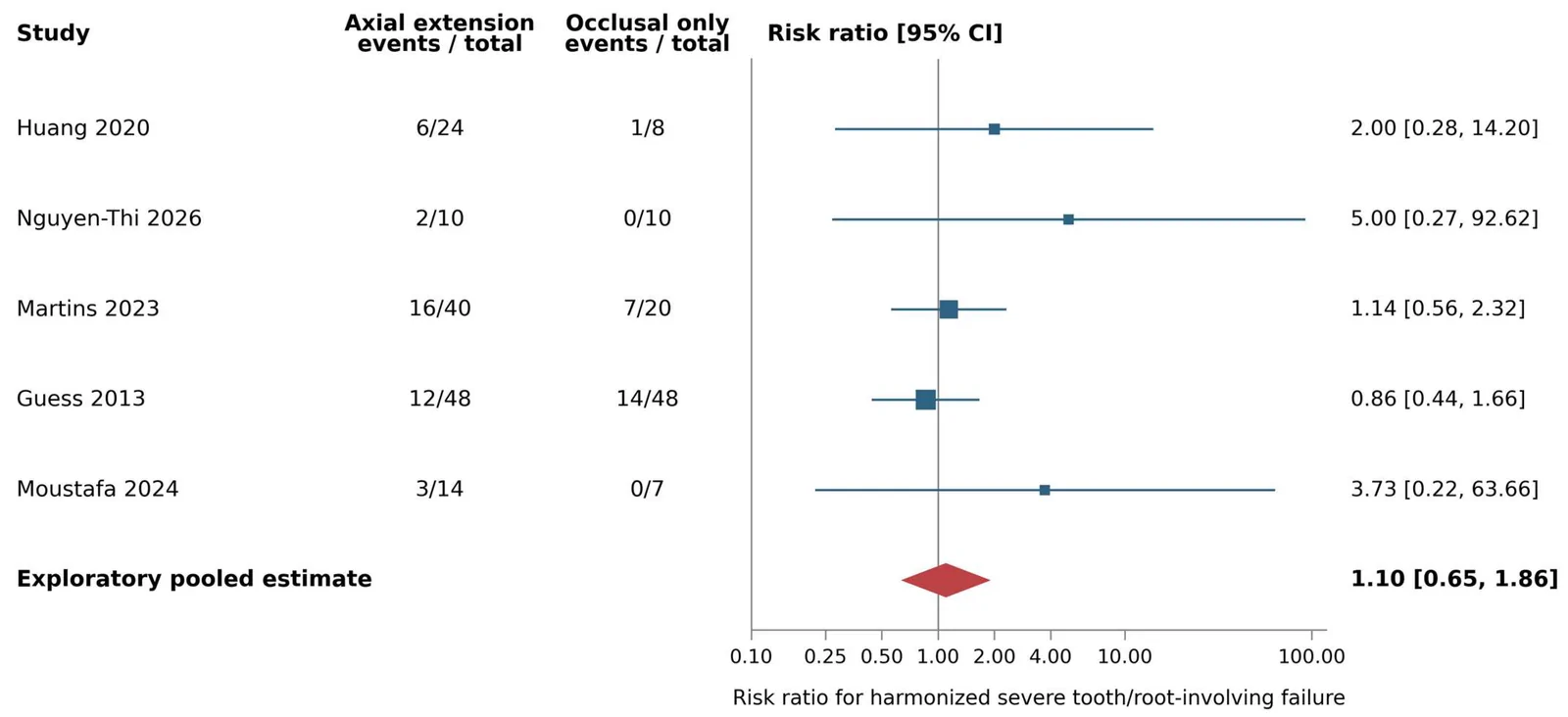
Study-specific event counts and exploratory random-effects risk-ratio analysis of review-defined severe failure: axial-extension versus occlusal-only restorations. Within each study, all eligible axial-extension arms with group-resolved events were combined; the pooled estimate therefore includes both single- and multi-surface designs. RR < 1 indicates fewer review-defined severe failures in the axial-extension group. The pooled estimate used REML with the Hartung–Knapp adjustment; a 0.5 continuity correction was applied only to studies containing a zero cell. Because source definitions differed and events were sparse, the pooled estimate is exploratory [8,26,27,28,30].

Five studies contributed group-resolved events to the axial-extension versus occlusal-only synthesis. Within each study, all eligible axial-extension arms with group-resolved events were combined; the pooled estimate therefore includes both single- and multi-surface designs and is exploratory. Exact events and totals are presented in Figure 3 and Table 5. The pooled RR was 1.10 (95% CI 0.65 to 1.86; I^2^ = 0%; τ^2^ = 0). The absence of detected heterogeneity should not be interpreted as evidence of homogeneity of source definitions or underlying effects because the synthesis was small and several studies contained sparse events. The confidence interval remains compatible with appreciably fewer or more review-defined severe failures. An equal-effects Mantel–Haenszel sensitivity analysis based directly on the cell counts and without continuity correction in the pooled estimate yielded RR 1.19 (95% CI 0.75 to 1.90), without changing the interpretation.

For comparisons with crowns, Huang et al. [27] reported 6/24 versus 0/8 severe events, while Nguyen-Thi et al. [30] reported 2/10 versus 6/10. Sayed et al. [21] did not provide group-resolved events. With only two usable and discordant comparisons, no pooled crown estimate was calculated.

A sensitivity analysis restricted to a shared explicit restorability definition could not be performed because the source criteria were not sufficiently consistent. Accordingly, this review-defined outcome should not be interpreted as necessarily catastrophic or clinically non-restorable.

### 3.8. Failure-Mode Harmonization and Data Integrity

Gierthmuehlen et al. explicitly stated that the control groups in their study originated from the earlier experiment reported by Guess et al. and were tested using an identical setup [8,22]. To avoid double-counting these data, Guess et al. was retained as the earlier primary report, and Gierthmuehlen et al. was summarized narratively. Two corresponding group means were identical, whereas a third nominally comparable value differed; therefore, the exact extent of data reuse could not be reconstructed, and residual uncertainty remains.

### 3.9. Confidence in Interpretation and Applicability

No formal overall certainty grade was assigned. Table 6 separately presents the laboratory evidence base, statistical precision and consistency, and methodological limitations and clinical applicability; these dimensions were not collapsed into a single score.

## 4. Discussion

### 4.1. Principal Findings

The present laboratory findings should be interpreted alongside, but not replaced by, clinical evidence on the broader family of bonded partial-coverage restorations. Long-term cohorts have reported high survival for lithium-disilicate occlusal onlays and other defect-specific partial-coverage restorations [10,11,12]. However, these clinical datasets do not compare the same restoration with and without an axial extension. They therefore support the clinical viability of conservative adhesive treatment in selected patients, but cannot resolve whether axial extension itself is beneficial, neutral, or harmful. This distinction is important because preparation design can influence fracture resistance [16], while single-load-to-failure testing does not reproduce the fatigue processes responsible for many clinical ceramic failures [31,32].

This morphology-based review found insufficient evidence to determine whether the evaluated axial-extension designs differ from occlusal-only restorations with respect to failure load or the occurrence of review-defined severe failure. This should not be interpreted as evidence that axial extension has no effect. For the primary failure load analysis, the 95% confidence interval extended from a 195 N disadvantage to a 149 N advantage, and the prediction interval was wider still. For review-defined severe failure, the interval ranged from a 35% relative reduction to an 86% relative increase. Both outcomes therefore remain uncertain.

In the broad all-eligible-geometry analysis, an arm-level model treating comparisons with a shared control as independent would have inflated the nominal number of effects from five study-level estimates to 13 arm-level comparisons. Group-level data were therefore checked against the primary reports; the final study-level analyses used the 833 N table value for Guess et al., with the discrepant 883 N abstract value evaluated separately in a sensitivity analysis, and incorporated corrected comparator values and Mode IV counts for Martins et al. and corrected denominators for Moustafa et al.

The comparison with full-coverage crowns is even less informative. Only three studies were available; their effects pointed in different directions, and they were clinically and technically heterogeneous. They were therefore not pooled, and their study-specific findings cannot support equivalence or non-inferiority.

### 4.2. Why the Available Evidence Does Not Fully Isolate the Effect of Axial Extension

Axial extension was rarely the only design feature that varied across the evidence base. Studies used occlusal reductions or restoration thicknesses from 0.5 to 2.5 mm, axial reductions or finish-line depths from 0.3 to 1.0 mm, and different intracoronal-box geometries, substrates, and numbers of axially extended surfaces. Within the five studies contributing to the primary single-surface axial-extension versus occlusal-only comparisons, occlusal dimensions were generally matched between the relevant arms, which supports within-study comparability for this dimension. Across studies, however, the axial depth, location of the finish line, and amount of remaining enamel differed. The primary meta-analysis therefore provides an exploratory average across various single-surface axial-extension designs and depth protocols; it cannot identify an optimal preparation depth or separate the effect of depth from material, substrate, tooth type, ageing, and loading protocols.

Preparation depth should consequently be treated as a design and comparability variable, not as a stand-alone quality score. A deeper or shallower preparation is not inherently a source of bias if it is standardized, measured, and matched between the relevant arms. Bias or interpretive concern arises when dimensions are insufficiently reported, not verified after preparation, or changed together with the experimental factor so that their effects cannot be separated.

More broadly, the mechanical performance of an adhesively retained restoration should be regarded as a system-level outcome rather than as a consequence of preparation geometry alone. Across experimental restorative models, fatigue performance, fracture behaviour, and load-bearing response have varied with restorative material [33,34], restoration thickness and bonding substrate [22,35,36], bonding method [37], and preparation design [22,38,39,40]. Immediate dentine sealing has also affected performance in some experimental models [41,42]. The included reports used conventional adhesive resin cements, self-adhesive dual-cure cements, or heated restorative composite, together with different substrate and intaglio treatments. Because none of the direct comparisons varied these factors independently of preparation geometry, their contributions cannot be estimated from this review and remain possible sources of between-study variation.

Cusp function is a plausible modifier but cannot be established from these data. In maxillary posterior teeth, the palatal cusp is generally load-bearing, whereas, in mandibular posterior teeth, the buccal cusp is generally load-bearing [43]. Consequently, a buccal extension does not represent the same functional situation in both arches. The available observations in Huang et al. and Moustafa et al. are based on only two studies and are confounded by arch, tooth type, material, and loading protocol [26,27]. They may inform the design of future experiments but should not support clinical recommendations.

Loading and ageing conditions are also components of the measured outcome rather than neutral methodological details. Within the included evidence base, most studies used a vertical or approximately axial static load, whereas Martins et al. applied load at 135° to the long axis, and several ageing protocols introduced lateral movement before static testing [28]. Indenter diameter, contact position, and periodontal-ligament simulation also varied or were incompletely reported, potentially affecting stress concentration and crack propagation [31]. Related occlusal-veneer studies indicate that thermomechanical fatigue may affect survival and fracture strength [44] while values obtained under lateral static loading are not directly comparable with axial-loading results [45].

Absolute load-to-failure values should not be translated into a clinical safety threshold. Static loading to fracture does not reproduce repeated multidirectional loading, moisture, temperature, pH, adhesive degradation, biological complications, or patient-level parafunction. The fact that a specimen failed above a commonly cited masticatory-force range does not demonstrate clinical adequacy.

Taken together, the wider experimental literature provides relevant mechanistic context, although differences in restorative architecture, substrate, preparation, and testing protocols limit direct quantitative extrapolation to axial-extension designs [18]. Nevertheless, the evidence supports the view that measured performance reflects interactions among material-, geometry-, interface-, and loading-related factors. Consequently, the pooled estimate should be interpreted as an average across heterogeneous experimental systems and cannot determine how these co-determinants modify the effect of axial extension.

### 4.3. Failure Mode and Restorability

Failure mode may be more clinically interpretable than peak load only when definitions are consistent. The included studies used related but not identical failure-mode classification schemes, and some descriptions combined restoration fracture, tooth fracture, pulpal involvement, extension below the cementoenamel junction, and root fracture. These events are not automatically equivalent. The review therefore used a review-defined severe-failure category, comprising fractures extending below the cementoenamel junction or involving the root, and excluded data that could not be assigned at group level. This category was not treated as equivalent to a uniform clinical judgment of non-restorability.

The exploratory pooled RR for this review-defined category was close to 1, but its confidence interval was not narrow. Sparse events and zero-event cells further limited precision. The appropriate conclusion is that the direction and magnitude of any effect remain uncertain—not that the designs have the same failure pattern.

Future experiments should report both the original fracture morphology and a binary restorability outcome defined before testing. Photographs or micro-CT documentation, blinded assessment, and explicit criteria for repairability, endodontic involvement, and subgingival extension could improve the reliability of failure-mode synthesis.

### 4.4. Nomenclature and Cross-Report Data Overlap

The intervention could not be identified reliably by the word “vonlay.” A morphology-based definition recovered studies using several unrelated labels and excluded nominal vonlay reports that did not have the required geometry. A standardized preparation diagram and minimum geometry dataset should accompany future publications.

Gierthmuehlen et al. reported that the control groups originated from the earlier Guess et al. experiment [8,22]. Because the extent of the shared data could not be reconstructed reliably, the Guess et al. report was retained in the quantitative synthesis as the earlier report containing the directly extracted comparator data, whereas the Gierthmuehlen et al. report was summarized narratively; residual uncertainty remains. Primary reports should identify reused specimens or datasets explicitly, and systematic reviewers should compare descriptive statistics before counting reports as independent.

### 4.5. Strengths and Limitations

Strengths of the review include a morphology-based eligibility definition, independent study selection, data extraction and appraisal, separate consideration of failure load and failure morphology, explicit definition of the review-defined severe-failure category, verification of group-level data against the primary reports, and explicit control of shared-comparator dependence. The ratio-of-means analysis complemented the absolute mean-difference analysis, while the leave-one-study-out and Mantel–Haenszel sensitivity analyses assessed the robustness of the failure-load and sparse-event results, respectively.

The review also has important limitations. Only eight studies were eligible, and only five contributed to the primary quantitative comparison. The included experiments also had small groups, incomplete reporting of randomization, no reported outcome-assessor blinding, and inconsistent verification of achieved preparation dimensions. The search concerns a poorly indexed intervention, making incomplete retrieval possible despite citation searching. The review was not prospectively registered, and no formal a priori protocol was prepared; the review-defined failure category, quantitative-analysis hierarchy, and sensitivity analyses were not prospectively specified. Some numerical values were manually estimated from published figures; one study had internally inconsistent failure-mode reporting, the study authors were not contacted for clarification, and the exact extent of cross-report control-data reuse could not be reconstructed. Within-study aggregation combined intervention arms that differed in the surface and extent of extension; this avoided repeated use of shared comparators but limits design-specific conclusions. Finally, all evidence was in vitro and cannot establish clinical longevity, biological safety, or patient-reported outcomes.

### 4.6. Implications for Research and Practice

Future studies should isolate the presence, surface, or extent of axial extension as the planned experimental factor and should use a priori sample-size calculations. At minimum, authors should report: tooth type and arch; random-sequence generation and allocation procedures; standardized diagrams and photographs of the preparation; occlusal reduction at fissures and cusp tips; axial reduction at the cervical and occlusal parts of the extension; finish-line type, depth and distance from the cementoenamel junction; restoration thickness at the same reference points; functional status of the extended cusp; remaining enamel and dentin thickness; presence, depth and width of any intracoronal box; restorative material; adhesive/luting protocol; periodontal-ligament simulation; ageing and thermocycling; indenter diameter, contact points and loading angle; blinded outcome assessment; all exclusions after allocation; group-level means, standard deviations and raw event counts; and prespecified restorability criteria. Digital or silicone-index verification of the achieved, rather than merely intended, dimensions would improve reproducibility. Testing buccal and lingual extensions within the same arch and tooth type could help evaluate the cusp-function hypothesis [43].

For clinical practice, this review does not show that the evaluated axial-extension designs should routinely be used to increase strength or to prevent review-defined severe failure. It also does not exclude the possibility of harm. Clinical selection may instead be guided by the actual defect, the need to replace a missing axial surface, margin placement, available enamel, restorative space, emergence profile, and aesthetic requirements. These are clinical rationales, not conclusions of the meta-analysis.

## 5. Conclusions

The current in vitro evidence base is too small, heterogeneous, and imprecise to determine reliably whether the evaluated axial-extension designs differ from occlusal-only restorations with respect to failure load or the occurrence of review-defined severe failure. This evidence is also indirect with respect to clinical performance. The pooled estimates represent average effects across heterogeneous combinations of geometry, material, and testing conditions rather than the effect of one standardized axial-extension design. In the primary single-surface failure-load comparison, the RoM was 0.98 (95% CI 0.88 to 1.09) and the MD was −23 N (95% CI −195 to 149). In the exploratory severe-failure synthesis, which combined all eligible axial-extension geometries with group-resolved events within studies, the pooled RR was 1.10 (95% CI 0.65 to 1.86). These intervals exclude neither important benefit nor important harm. The three heterogeneous crown comparisons were not pooled and remain inconclusive. The review’s main contribution is therefore methodological: it defines the intervention by geometry, identifies dependence and reporting problems in the evidence base, and specifies variables that future experiments should address before clinically meaningful comparisons are possible.

## Figures and Tables

**Table 1:** Characteristics of the included studies and review-relevant experimental arms. AE, axial extension; OO, occlusal-only; LD, lithium disilicate; ZLS, zirconia-reinforced lithium silicate; PICN, polymer-infiltrated ceramic network; IDS, immediate dentin sealing; MOD, mesio-occluso-distal; NR, not reported; M, million. OF, POF, POFP, O, OV, OVP, and VL are report-specific arm labels; the corresponding preparation geometry is described in the adjacent column. “Primary” denotes the single-surface axial-extension comparison, whereas “broad” includes all eligible axial-extension geometries.

Study	Teeth/Material	Review-Relevant Arms and Analysed n	Preparation Design	Ageing and Static Loading	Adhesive or Luting Protocol
Guess 2013 [8]	Maxillary premolars; pressed LD	AE complete veneer: 3 arms × 16; OO onlay: 3 arms × 16	Common MOD inlay preparation in all arms; labial extension in complete-veneer arms; three matched thickness strata	1.2 M cycles + thermocycling; axial	Syntac Classic + Variolink II
Huang 2020 [27]	Maxillary premolars; pressed LD	Primary AE OF: 8; broad adds POF/POFP: n = 8 each; OO O: 8; crown: 8	Lingual extension, with or without proximal extension; no box	20,000 cycles; axial	Multilink Primer A/B + Multilink Automix
Gierthmuehlen 2022 [22]	Maxillary premolars; pressed LD	AE full veneer: 5 × 12; previously reported OO control data: 3 arms × 16; narrative only	Labial extension; five full-veneer groups differing in thickness and bonding substrate	1.2 M cycles + thermocycling; axial	Syntac Classic + Variolink II
Sayed 2022 [21]	Maxillary premolars; CAD/CAM LD	AE vonlay group B: 10; crown: 10	Labial extension with functional-cusp coverage; box	Thermocycling; axial; indenter report inconsistent	Dual-cure self-adhesive resin cement; brand NR
Martins 2023 [28]	Third molars; semi-direct resin composite	Primary AE OV: 20; broad adds OVP: 20; OO O: 20	Buccal or multi-surface extension; no box	0 or 500,000 cycles; 135 degrees	Single Bond Universal + RelyX ARC
Moustafa 2024 [26]	Mandibular molars; LD	AE buccal: n = 7; AE lingual: n = 8; OO: n = 7 ^1^	Single buccal or lingual extension; no box	150,000 cycles + thermocycling; vertical loading; orientation to tooth axis unclear; indenter dimensions/contact NR	Multilink Speed
Al-Mazzaly 2026 [29]	Maxillary premolars; LD, ZLS, zirconia, PICN	AE: four material arms, n = 14 each; no preparation-design comparator	Buccal and palatal extension; MOD box	500,000 cycles; direction ambiguously reported	Dual-cure self-adhesive resin cement; brand NR
Nguyen-Thi 2026 [30]	Maxillary premolars; 3D-printed composite	AE VL: 10; OO OV: 10; crown: 10	Buccal extension; no box	Seven-day storage; no cycling; axial	IDS + Single Bond Universal; heated Filtek Z250

^1^ Sample sizes in this column refer to the failure-load analysis. For failure-mode assessment in Moustafa et al., the published figure accounted for seven specimens in each group (AE total, n = 14; OO, n = 7).

**Table 2:** Reported preparation dimensions and within-study comparability. Values are presented as described in the primary reports; occlusal reduction, restoration thickness, axial reduction, and finish-line depth are related but not interchangeable quantities. CEJ, cementoenamel junction; MOD, mesio-occluso-distal.

Study	Reported Occlusal Reduction or Restoration Thickness	Reported Axial Reduction or Finish-Line Depth	Intracoronal Box	Within-Study Comparability
Guess 2013 [8]	2.0, 1.0 or 0.5 mm, according to thickness stratum	Labial chamfer 0.8, 0.6 or 0.4 mm	Yes, in all arms; common MOD preparation with a 3-mm-deep × 2-mm-wide isthmus; proximal boxes ended 1 mm above the CEJ	Common MOD preparation and occlusal depth matched within each thickness stratum; the labial chamfer distinguished the complete-veneer arms
Huang 2020 [27]	0.7 mm at fissures; 1.0 mm at cusps	Lingual/proximal reduction 0.5–1.0 mm; 0.5-mm chamfer 1 mm above CEJ	No	Occlusal depth matched; number and position of axial/proximal surfaces varied
Gierthmuehlen 2022 [22]	Reported occlusal thickness 1.5, 1.0 or 0.5 mm	Reported labial/proximal thickness 0.8, 0.6 or 0.4 mm	No in test arms; controls had a 3-mm-deep × 2-mm-wide box	Thickness strata were standardized; the control groups differed in intracoronal-box design and bonding substrate; narrative synthesis only
Sayed 2022 [21]	2-mm functional-cusp reduction	0.5-mm labial chamfer	Yes; 2 mm deep and half the buccolingual width	No occlusal-only comparator; comparison with the crown involved multiple geometric differences
Martins 2023 [28]	1-mm occlusal reduction	Buccal extension ≤ 1 mm; circumferential chamfer extended 1 mm occlusocervically	No	Occlusal depth matched; extent and number of axial surfaces varied
Moustafa 2024 [26]	2.5 mm occlusal reduction	0.3 mm cervically and 0.5 mm occlusally; finish line 1 mm above CEJ	No	Occlusal depth was matched; the groups differed in the presence and surface of the axial extension
Al-Mazzaly 2026 [29]	MOD cavity depth 2 mm; cusp reduction 1.0 mm buccal and 1.5 mm palatal	0.5-mm buccal chamfer; 1-mm palatal shoulder	Yes (MOD)	Same preparation across material groups; no occlusal-only design comparator
Nguyen-Thi 2026 [30]	1.0–1.2 mm	Veneerlay buccal chamfer 0.8–1.0 mm	No	Occlusal depth was matched between the overlay and veneerlay groups; axial extent differed among the overlay, veneerlay and crown groups

**Table 3:** Author-reported terminology and morphological eligibility features of the included axial-extension restorations.

Study	Author Term	Morphological Reason for Eligibility
Guess 2013 [8]	Complete veneer	Complete occlusal coverage + labial axial finish line
Huang 2020 [27]	OF/POF/POFP occlusal veneers	Complete occlusal coverage + palatal/lingual extension
Gierthmuehlen 2022 [22]	Posterior minimally invasive full veneer	Complete occlusal coverage + labial extension
Sayed 2022 [21]	Vonlay	Complete occlusal coverage + labial extension
Martins 2023 [28]	Occlusal veneer/overlay	Complete occlusal coverage + axial extension(s)
Moustafa 2024 [26]	Occlusal veneer with buccal/lingual extension	Complete occlusal coverage + one axial extension
Al-Mazzaly 2026 [29]	Vonlay	Complete occlusal coverage + buccal and palatal axial extension
Nguyen-Thi 2026 [30]	Veneerlay	Complete occlusal coverage + buccal extension

**Table 4:** Domain-level RoBDEMAT profile and supplementary QUIN summary. Within-domain item order: D1, control group (C)/randomization (R)/sample-size rationale (N); D2, specimen/material standardization (S)/identical experimental conditions (C); D3, standardized testing (T)/blinding (B); D4, statistical analysis (A)/outcome reporting (O). SR, sufficiently reported; IR, insufficiently reported; NR, not reported. Control adequacy was judged relative to the stated experimental aim and did not require an intact-tooth group when a suitable active or existing-standard comparator was present.

Study	D1 Planning/Allocation (C/R/N)	D2 Preparation (S/C)	D3 Outcome (T/B)	D4 Analysis/Reporting (A/O)	QUIN Score/Category
Al-Mazzaly 2026 [29]	SR/IR/SR	SR/IR	IR/NR	SR/SR	62.5%/Medium risk
Guess 2013 [8]	SR/NR/NR	SR/SR	IR/NR	SR/IR	50.0%/Medium risk
Gierthmuehlen 2022 [22]	IR/IR/NR	IR/IR	SR/NR	SR/IR	50.0%/Medium risk
Huang 2020 [27]	SR/IR/IR	IR/SR	SR/NR	SR/IR	66.7%/Medium risk
Nguyen-Thi 2026 [30]	SR/IR/IR	IR/IR	SR/NR	SR/IR	70.8%/Low risk
Sayed 2022 [21]	SR/NR/NR	IR/IR	IR/NR	IR/IR	29.2%/High risk
Martins 2023 [28]	SR/IR/IR	IR/SR	IR/NR	SR/IR	66.7%/Medium risk
Moustafa 2024 [26]	SR/SR/SR	SR/SR	IR/NR	IR/IR	62.5%/Medium risk

**Table 5:** Harmonization of the review-defined severe-failure category and comparison-arm allocation. CEJ, cementoenamel junction; NA, not applicable; NR, not reported or not reconstructable.

Study	Source Category Mapped to Severe Event	Axial-Extension Events/Total	Occlusal-Only Events/Total	Crown Events/Total
Guess 2013 [8]	Type IV; root-involving	12/48	14/48	NA
Huang 2020 [27]	Mode IV below CEJ	6/24	1/8	0/8
Gierthmuehlen 2022 [22]	Type IV tooth/root involvement	NR	NR	NA
Sayed 2022 [21]	Aggregate failure percentages	NR	NA	NR
Martins 2023 [28]	Score/Mode IV	16/40	7/20	NA
Moustafa 2024 [26]	Mode IV below cervical line/root	3/14	0/7	NA
Al-Mazzaly 2026 [29]	Mode IV; root-involving	NR	NA	NA
Nguyen-Thi 2026 [30]	Mode 4; root-involving	2/10	0/10	6/10

**Table 6:** Outcome-level interpretation of the laboratory evidence and its clinical applicability. AE, axial-extension design; OO, occlusal-only restoration.

Outcome	Laboratory Evidence Base	Statistical Precision and Consistency	Methodological Limitations	Clinical Applicability
Failure load: AE vs. OO	Five independent studies; single-surface primary analysis plus broad sensitivity analysis	Principal CIs and prediction intervals crossed the null; the leave-one-study-out result was sensitive to omission of Guess	Different teeth, materials, preparation dimensions, luting, ageing and loading	Insufficient to determine clinical benefit or harm; the estimate does not represent one standardized design
Review-defined severe failure: AE vs. OO	Five group-resolved studies; harmonized review-defined outcome	Sparse events, wide study-specific CIs and exploratory pooled RR	Non-identical definitions, zero cells and no common prospective restorability criterion	Cannot establish clinical restorability, clinical failure or patient-important harm
Failure load: AE vs. crown	Three heterogeneous comparisons; narrative synthesis	Estimates pointed in opposite directions; no pooled estimate	Different materials, geometries, ageing procedures and loading protocols	Inconclusive; no inference of superiority, equivalence or non-inferiority
Severe failure: AE vs. crown	Two usable group-resolved comparisons	Discordant event patterns; no pooled estimate	One additional study lacked group-resolved data	Insufficient for a comparative clinical conclusion

## Data Availability

The original contributions presented in the study are included in the article/Supplementary Material, further inquiries can be directed to the corresponding authors.

## Supplementary Materials

The following supporting information can be downloaded at: https://www.mdpi.com/article/10.3390/jfb17100497/s1, PRISMA 2020 Checklist; Table S1. Complete database-specific search strategies; Table S2. Detailed risk-of-bias assessments using RoBDEMAT and QUIN; Table S3. Reports excluded after full-text assessment and primary reasons for exclusion; Table S4. Study-level outcome data contributing to the quantitative syntheses; Data S1, study-level dataset and audit trail; File S1, reproducible R code.

## Abbreviations

The following abbreviations are used in this manuscript:

PRISMA	Preferred Reporting Items for Systematic Reviews and Meta-Analyses
MD	Mean difference
CI	Confidence interval
I^2^	I-squared heterogeneity statistic
RR	Risk ratio
MH	Mantel–Haenszel
QUIN	Quality Assessment Tool for In Vitro Studies
SWiM	Synthesis Without Meta-analysis
GRADE	Grading of Recommendations Assessment, Development and Evaluation
RoBDEMAT	Risk of Bias tool for DEntal MATerials research
REML	Restricted maximum likelihood
CEJ	Cementoenamel junction
CAD/CAM	Computer-aided design/computer-aided manufacturing
PICN	Polymer-infiltrated ceramic network
ZLS	Zirconia-reinforced lithium silicate

## References

[1] Van’t Spijker A., Rodriguez J.M., Kreulen C.M., Bronkhorst E.M., Bartlett D.W., Creugers N.H. (2009). Prevalence of tooth wear in adults. Int. J. Prosthodont..

[2] Bartlett D.W., Lussi A., West N.X., Bouchard P., Sanz M., Bourgeois D. (2013). Prevalence of tooth wear on buccal and lingual surfaces and possible risk factors in young European adults. J. Dent..

[3] Edelhoff D., Sorensen J.A. (2002). Tooth structure removal associated with various preparation designs for posterior teeth. Int. J. Periodontics Restor. Dent..

[4] Magne P., Belser U. (2002). Bonded Porcelain Restorations in the Anterior Dentition: A Biomimetic Approach.

[5] Opdam N., Frankenberger R., Magne P. (2016). From ‘Direct Versus Indirect’ Toward an Integrated Restorative Concept in the Posterior Dentition. Oper. Dent..

[6] Vailati F., Belser U.C. (2008). Full-mouth adhesive rehabilitation of a severely eroded dentition: The three-step technique. Part 1. Eur. J. Esthet. Dent..

[7] Vailati F., Belser U.C. (2008). Full-mouth adhesive rehabilitation of a severely eroded dentition: The three-step technique. Part 2. Eur. J. Esthet. Dent..

[8] Guess P.C., Schultheis S., Wolkewitz M., Zhang Y., Strub J.R. (2013). Influence of preparation design and ceramic thicknesses on fracture resistance and failure modes of premolar partial coverage restorations. J. Prosthet. Dent..

[9] McLaren E.A., Figueira J., Goldstein R.E. (2015). Vonlays: A conservative esthetic alternative to full-coverage crowns. Compend. Contin. Educ. Dent..

[10] Edelhoff D., Güth J.F., Erdelt K., Brix O., Liebermann A. (2019). Clinical performance of occlusal onlays made of lithium disilicate ceramic in patients with severe tooth wear up to 11 years. Dent. Mater..

[11] Malament K.A., Margvelashvili-Malament M., Natto Z.S., Thompson V., Rekow D., Att W. (2021). Comparison of 16.9-year survival of pressed acid etched e.max lithium disilicate glass-ceramic complete and partial coverage restorations in posterior teeth: Performance and outcomes as a function of tooth position, age, sex, and thickness of ceramic material. J. Prosthet. Dent..

[12] Margvelashvili-Malament M., Thompson V., Polyakov V., Malament K.A. (2025). Over 14-year survival of pressed e.max lithium disilicate glass-ceramic complete and partial coverage restorations in patients with severe wear: A prospective clinical study. J. Prosthet. Dent..

[13] Charoenporn W., Sornsuwan T., Sae-Lee D., Amornvit P., Chaijareenont P., Rungsiyakull P. (2024). Evaluating fatigue resistance in occlusal veneers: A comparative study of processing techniques and material thickness of lithium disilicate (IPS e.max Press vs. IPS e.max CAD). BMC Oral Health.

[14] Czechowski Ł., Dejak B., Konieczny B., Krasowski M. (2023). Evaluation of Fracture Resistance of Occlusal Veneers Made of Different Types of Materials Depending on Their Thickness. Materials.

[15] Dellazzana F.Z., Souza L.F.B., Silva G.R., Freitas J.S., dos Santos S.S., Pereira G.K.R., Rodrigues C.d.S., May L.G. (2026). Fatigue behavior of dental ceramics for occlusal veneers: Impact of the material thickness. J. Mech. Behav. Biomed. Mater..

[16] Sirous S., Navadeh A., Ebrahimgol S., Atri F. (2022). Effect of preparation design on marginal adaptation and fracture strength of ceramic occlusal veneers: A systematic review. Clin. Exp. Dent. Res..

[17] Ladino L., Sanjuan M.E., Valdez D.J., Eslava R.A. (2021). Clinical and Biomechanical Performance of Occlusal Veneers: A Scoping Review. J. Contemp. Dent. Pract..

[18] Albelasy E.H., Hamama H.H., Tsoi J.K.H., Mahmoud S.H. (2020). Fracture resistance of CAD/CAM occlusal veneers: A systematic review of laboratory studies. J. Mech. Behav. Biomed. Mater..

[19] Page M.J., McKenzie J.E., Bossuyt P.M., Boutron I., Hoffmann T.C., Mulrow C.D., Shamseer L., Tetzlaff J.M., Akl E.A., Brennan S.E. (2021). The PRISMA 2020 statement: An updated guideline for reporting systematic reviews. Int. J. Surg..

[20] Campbell M., McKenzie J.E., Sowden A., Katikireddi S.V., Brennan S.E., Ellis S., Hartmann-Boyce J., Ryan R., Shepperd S., Thomas J. (2020). Synthesis without meta-analysis (SWiM) in systematic reviews: Reporting guideline. BMJ.

[21] Sayed E., Waz S., Mohamed M. (2022). Influence of different vonlay preparation designs on fracture resistance. Egypt. Dent. J..

[22] Gierthmuehlen P.C., Jerg A., Fischer J.B., Bonfante E.A., Spitznagel F.A. (2022). Posterior minimally invasive full-veneers: Effect of ceramic thicknesses, bonding substrate, and preparation designs on failure-load and -mode after fatigue. J. Esthet. Restor. Dent..

[23] Delgado A.H., Sauro S., Lima A.F., Loguercio A.D., Della Bona A., Mazzoni A., Collares F.M., Staxrud F., Ferracane J., Tsoi J. (2022). RoBDEMAT: A risk of bias tool and guideline to support reporting of pre-clinical dental materials research and assessment of systematic reviews. J. Dent..

[24] Sheth V.H., Shah N.P., Jain R., Bhanushali N., Bhatnagar V. (2024). Development and validation of a risk-of-bias tool for assessing in vitro studies conducted in dentistry: The QUIN. J. Prosthet. Dent..

[25] Viechtbauer W. (2010). Conducting Meta-Analyses in R with the metafor Package. J. Stat. Softw..

[26] Moustafa H.H. (2024). Assessment of fracture resistance of occlusal veneers with buccal or lingual extensions compared to conventional occlusal veneers restoring worn molars after thermo-mechanical aging. MSA Dent. J..

[27] Huang X., Zou L., Yao R., Wu S., Li Y. (2020). Effect of preparation design on the fracture behavior of ceramic occlusal veneers in maxillary premolars. J. Dent..

[28] Martins W.F., Coelho C.S.S., Amaral F., França F.M.G., Turssi C.P., Cavalli V., Basting R.T. (2023). Fracture load and failure mode of semi-direct resin composite occlusal veneers: Influence of design and mechanical cycling. J. Mech. Behav. Biomed. Mater..

[29] Al-Mazzaly A.M., Abdel-Fattah W., Nasr D.M. (2026). Fracture resistance of ceramic vonlays fabricated from different CAD/CAM materials restoring premolars after cyclic loading (an in vitro study). BMC Oral Health.

[30] Nguyen-Thi T.L., Tran T.M., Tran-Thi H.P., Nguyen-Ho P.M., Do T.T., Dang M.H. (2026). Effect of preparation design on the fracture behavior of 3D-printed restorations in an in-vitro tooth wear model. BMC Oral Health.

[31] Pereira G.K.R., Pilecco R.O., Tribst J.P.M. (2026). Fatigue in Dental Ceramics: A Call for Standardized Testing and Emerging Manufacturing Technologies. Materials.

[32] Kelly J.R., Cesar P.F., Scherrer S.S., Della Bona A., van Noort R., Tholey M., Vichi A., Lohbauer U. (2017). ADM guidance-ceramics: Fatigue principles and testing. Dent. Mater..

[33] Magne P., Schlichting L.H., Maia H.P., Baratieri L.N. (2010). In vitro fatigue resistance of CAD/CAM composite resin and ceramic posterior occlusal veneers. J. Prosthet. Dent..

[34] Schlichting L.H., Maia H.P., Baratieri L.N., Magne P. (2011). Novel-design ultra-thin CAD/CAM composite resin and ceramic occlusal veneers for the treatment of severe dental erosion. J. Prosthet. Dent..

[35] Sasse M., Krummel A., Klosa K., Kern M. (2015). Influence of restoration thickness and dental bonding surface on the fracture resistance of full-coverage occlusal veneers made from lithium disilicate ceramic. Dent. Mater..

[36] Abu-Izze F.O., Ramos G.F., Borges A.L.S., Anami L.C., Bottino M.A. (2018). Fatigue behavior of ultrafine tabletop ceramic restorations. Dent. Mater..

[37] Krummel A., Garling A., Sasse M., Kern M. (2019). Influence of bonding surface and bonding methods on the fracture resistance and survival rate of full-coverage occlusal veneers made from lithium disilicate ceramic after cyclic loading. Dent. Mater..

[38] Gierthmuehlen P.C., Spitznagel F.A., Koschate M., Bonfante E.A., Prott L.S. (2024). Influence of ceramic thickness and dental substrate on the survival rate and failure load of non-retentive occlusal veneers after fatigue. J. Esthet. Restor. Dent..

[39] Angerame D., De Biasi M., Agostinetto M., Franzò A., Marchesi G. (2019). Influence of preparation designs on marginal adaptation and failure load of full-coverage occlusal veneers after thermomechanical aging simulation. J. Esthet. Restor. Dent..

[40] Velho H.C., Dapieve K.S., Grassi E.D.A., Borges A.L.S., de Melo Marinho R.M., Pereira G.K.R., Venturini A.B., Valandro L.F. (2023). Fatigue behavior, failure mode, and stress distribution of occlusal veneers: Influence of the prosthetic preparation cusp inclinations and the type of restorative material. Clin. Oral Investig..

[41] Mueller B., Pilecco R.O., Valandro L.F., Ruschel V.C., Pereira G.K.R., Bernardon J.K. (2023). Effect of immediate dentin sealing on load-bearing capacity under accelerated fatigue of thin occlusal veneers made of CAD-CAM glass-ceramic and resin composite material. Dent. Mater..

[42] Yazigi C., Kern M., Chaar M.S. (2017). Influence of various bonding techniques on the fracture strength of thin CAD/CAM-fabricated occlusal glass-ceramic veneers. J. Mech. Behav. Biomed. Mater..

[43] Khers S.C., Carpenter C.W., Vetter J.D., Staley R.N. (1990). Anatomy of cusps of posterior teeth and their fracture potential. J. Prosthet. Dent..

[44] Al-Akhali M., Kern M., Elsayed A., Samran A., Chaar M.S. (2019). Influence of thermomechanical fatigue on the fracture strength of CAD-CAM-fabricated occlusal veneers. J. Prosthet. Dent..

[45] Zamzam H., Olivares A., Fok A. (2021). Load capacity of occlusal veneers of different restorative CAD/CAM materials under lateral static loading. J. Mech. Behav. Biomed. Mater..

