# Supplementary material for: Failure Load and Failure Modes of Indirect Posterior Restorations Combining Occlusal Coverage with Axial Extension: A Systematic Review and Exploratory Meta-Analysis of In Vitro Studies
Source: J Funct Biomater. 2026 Oct 1;17(10):497. doi: 10.3390/jfb17100497 (PMC13705702; doi:10.3390/jfb17100497)
Supplement: Supplementary file 1 [file jfb-17-00497-s001.zip › PRISMA_2020_Checklist.pdf]

PRISMA 2020 Checklist

Completed for: Failure load and failure modes of indirect posterior restorations combining occlusal coverage with axial extension: a systematic review and exploratory meta-analysis of in vitro studies

Completion note. Locations are given by manuscript section/table/figure so they remain valid if pagination changes during journal production.

Section and topic	Item #	Checklist item	Location where item is reported
Title	1	Identify the report as a systematic review.	Title.
Abstract	2	See the PRISMA 2020 for Abstracts checklist.	Abstract: objectives, information sources and last database-consultation date, eligibility, study-selection and risk-of-bias methods, quantitative synthesis methods and results, limitations, interpretation, registration, and funding.
Rationale	3	Describe the rationale for the review in the context of existing knowledge.	Section 1, Introduction.
Objectives	4	Provide an explicit statement of the objective(s) or question(s) the review addresses.	Section 1, final paragraph.
Eligibility criteria	5	Specify the inclusion and exclusion criteria for the review and how studies were grouped for the syntheses.	Section 2.1; Section 2.5.
Information sources	6	Specify all databases, registers, websites, organisations, reference lists and other sources searched or consulted to identify studies. Specify the date when each source was last searched or consulted.	Section 2.2; Supplementary Table S1. The available documentation supports month-level, but not exact calendar-date, reporting for the original search rounds: the initial searches were conducted during May–June 2026 and the broadened searches in June 2026. Results from both rounds were combined and deduplicated before screening. Citation searching began after final database deduplication; individual discovery dates and the total number of cited or citing records inspected were not separately logged. All four databases were last checked on 22 July 2026; no record new to the review set was identified or added to screening, and the gross displayed yield of that check was not separately logged.
Search strategy	7	Present the full search strategies for all databases, registers and websites, including any filters and limits used.	Section 2.2; Supplementary Table S1: verbatim database-specific strategies for both rounds, retrieval counts, rationale, chronology, and overlap management.
Selection process	8	Specify the methods used to decide whether a study met the inclusion criteria of the review, including how many reviewers screened each record and each report retrieved, whether they worked independently, and if applicable, details of automation tools used in the process.	Section 2.3. Two reviewers screened independently. Titles, authors, and publication dates were visible; masking of journal names was not documented. Rayyan concealed each reviewer's eligibility decision until both decisions were completed. No screening occurred between search rounds.
Data collection process	9	Specify the methods used to collect data from reports, including how many reviewers collected data from each report, whether they worked independently, any processes for obtaining or confirming data from study investigators, and if applicable, details of automation tools used in the process.	Section 2.3: duplicate independent extraction, consensus/adjudication, graph-derived values flagged, and study authors were not contacted.

Section and topic	Item #	Checklist item	Location where item is reported
Data items	10a	List and define all outcomes for which data were sought. Specify whether all results that were compatible with each outcome domain in each study were sought and, if not, the methods used to decide which results to collect.	Sections 2.1 and 2.3.
Data items	10b	List and define all other variables for which data were sought. Describe any assumptions made about any missing or unclear information.	Section 2.3; Data S1: Data_dictionary, Corrections_log, Decision_timeline, Evidence_audit, and outcome-specific attrition fields.
Study risk of bias assessment	11	Specify the methods used to assess risk of bias in the included studies, including details of the tool(s) used, how many reviewers assessed each study and whether they worked independently, and if applicable, details of automation tools used in the process.	Section 2.4; Table 4; Supplementary Table S2. RoBDEMAT is domain based and unscored; QUIN is supplementary; control adequacy is aim-specific; funding is separate.
Effect measures	12	Specify for each outcome the effect measure(s) used in the synthesis or presentation of results.	Section 2.5. Failure load: RoM and MD co-primary for the single-surface quantitative comparison. Severe failure: exact events/totals primary, RR exploratory. Crown comparisons: study-specific MD/RR only.
Synthesis methods	13a	Describe the processes used to decide which studies were eligible for each synthesis.	Sections 2.1 and 2.5; Data S1 Analysis_plan. The narrative synthesis includes all eight studies; the five-study single-surface, broad sensitivity, crown-descriptive, and severe-failure sets have explicit arm rules.
Synthesis methods	13b	Describe any methods required to prepare the data for presentation or synthesis, such as handling of missing summary statistics or data conversions.	Sections 2.3 and 2.5; Data S1. Combining equations, shared-control handling, outcome-specific denominators, graph-derived data, and 833/883 N discrepancy are documented.
Synthesis methods	13c	Describe any methods used to tabulate or visually display results of individual studies and syntheses.	Section 2.5; Tables 1-6; Supplementary Table S4; Figures 2-3. Exact severe-failure counts precede the exploratory RR and crown effects are unpooled.
Synthesis methods	13d	Describe any methods used to synthesize results and provide a rationale for the choice(s). If meta-analysis was performed, describe the model(s), methods to identify the presence and extent of statistical heterogeneity, and software package(s) used.	Section 2.5; File S1. Structured narrative synthesis is primary. Post hoc single-surface random-effects REML/Hartung-Knapp ratio-of-means and mean-difference models are the primary quantitative analyses; the all-geometry model is a sensitivity analysis. The log ratio of means uses the explicit second-order correction in metafor (correct = TRUE); tau-squared, I-squared, and prediction intervals are reported.
Synthesis methods	13e	Describe any methods used to explore possible causes of heterogeneity among study results.	Section 2.5; Sections 3.2-3.5 and 4.2. No subgroup/meta-regression was attempted because k was small and candidate modifiers were confounded with study.
Synthesis methods	13f	Describe any sensitivity analyses conducted to assess robustness of the synthesized results.	Section 2.5; Sections 3.5-3.8; File S1. Broad all-geometry, leave-one-out, Guess 833/883 N, and sparse-event Mantel-Haenszel sensitivities are reproducible.
Reporting bias assessment	14	Describe any methods used to assess risk of bias due to missing results in a synthesis (arising from reporting biases).	Section 2.5: no formal small-study/reporting-bias assessment was undertaken because each synthesis included fewer than 10 studies.
Certainty assessment	15	Describe any methods used to assess certainty (or confidence) in the body of evidence for an outcome.	Section 2.4; Section 3.9; Table 6. No unsupported overall numeric certainty score was used; directness, precision, consistency, RoB, and clinical applicability are separated.
Study selection	16a	Describe the results of the search and selection process, from the number of records identified in the search to the number of studies included in the review, ideally using a flow diagram.	Section 3.1; Figure 1. The flow diagram distinguishes records, reports, and studies and identifies two unique additional reports advanced to full-text assessment after citation searching. The total number of cited or citing records inspected was not separately logged.

Section and topic	Item #	Checklist item	Location where item is reported
Study selection	16b	Cite studies that might appear to meet the inclusion criteria, but which were excluded, and explain why they were excluded.	Supplementary Table S3 (20 full-text reports excluded, with primary reasons and bibliographic citations).
Study characteristics	17	Cite each included study and present its characteristics.	Sections 3.2–3.3; Tables 1–3.
Risk of bias in studies	18	Present assessments of risk of bias for each included study.	Section 3.4; Table 4; Supplementary Table S2.
Results of individual studies	19	For all outcomes, present, for each study: (a) summary statistics for each group (where appropriate) and (b) an effect estimate and its precision, ideally using structured tables or plots.	Tables 1 and 5; Supplementary Table S4; Data S1; Figures 2-3. Group summaries, individual effects/CIs, and exact events/totals are provided.
Results of syntheses	20a	For each synthesis, briefly summarise the characteristics and risk of bias among contributing studies.	Sections 3.2-3.7; Tables 1-6. Geometry, material, adhesive/luting, ageing, loading, attrition, and RoB are described before pooled estimates.
Results of syntheses	20b	Present results of all statistical syntheses conducted. If meta-analysis was done, present for each the summary estimate and its precision and measures of statistical heterogeneity. If comparing groups, describe the direction of the effect.	Sections 3.5-3.8; Figures 2-3; Supplementary Table S4D. Single-surface and broad failure-load models, severe-failure exploratory RR, precision, tau-squared, I-squared, and prediction intervals are reported.
Results of syntheses	20c	Present results of all investigations of possible causes of heterogeneity among study results.	No formal subgroup/meta-regression because k = 5 and modifiers were study-confounded; rationale in Section 2.5 and cautious observations in Sections 3.5 and 4.2.
Results of syntheses	20d	Present results of all sensitivity analyses conducted to assess the robustness of the synthesized results.	Sections 3.5-3.8; Supplementary Table S4; File S1. Broad geometry, leave-one-out, Guess discrepancy, and Mantel-Haenszel sensitivities are reported.
Reporting biases	21	Present assessments of risk of bias due to missing results (arising from reporting biases) for each synthesis assessed.	Not formally assessed because all syntheses contained fewer than 10 studies; rationale reported in Section 2.5.
Certainty of evidence	22	Present assessments of certainty (or confidence) in the body of evidence for each outcome assessed.	Section 3.9; Table 6: outcome-specific laboratory directness, statistical precision/consistency, methodological limitations, and clinical applicability.
Discussion	23a	Provide a general interpretation of the results in the context of other evidence.	Sections 4.1–4.4.
Discussion	23b	Discuss any limitations of the evidence included in the review.	Sections 4.1-4.5: heterogeneity, confounding, static/fatigue limitations, sparse events, definition mismatch, overlap, and reporting limitations.
Discussion	23c	Discuss any limitations of the review processes used.	Section 4.5: retrospective registration, post hoc synthesis decisions, missing author contact, graph-derived values, and unresolved record limitations.
Discussion	23d	Discuss implications of the results for practice, policy, and future research.	Section 4.6.
Registration and protocol	24a	Provide registration information for the review, including register name and registration number, or state that the review was not registered.	Section 2, opening paragraph: retrospective OSF registration of the review methods, DOI 10.17605/OSF.IO/9PXE5.
Registration and protocol	24b	Indicate where the review protocol can be accessed, or state that a protocol was not prepared.	Section 2, opening paragraph: no formal protocol was prepared before data extraction.

Section and topic	Item #	Checklist item	Location where item is reported
Registration and protocol	24c	Describe and explain any amendments to information provided at registration or in the protocol.	Section 2.5; Section 4.5; Data S1 Decision_timeline and Corrections_log. Post hoc harmonization, arm rules, overlap handling, and the sensitivity hierarchy are dated or labelled by stage.
Support	25	Describe sources of financial or non-financial support for the review, and the role of the funders or sponsors in the review.	Funding statement: This research received no external funding.
Competing interests	26	Declare any competing interests of review authors.	Conflicts of Interest statement.
Availability of data, code and other materials	27	Report which data, code and other review materials are publicly available and where they can be found.	Data Availability Statement; Data S1 and File S1. The workbook contains raw inputs, formulas, analysis plan, decision timeline, evidence audit, dictionary, and corrections; code writes result tables, plots, model objects, and session information.

Source: Page MJ, McKenzie JE, Bossuyt PM, Boutron I, Hoffmann TC, Mulrow CD, et al. The PRISMA 2020 statement: an updated guideline for reporting systematic reviews. BMJ. 2021;372:n71. doi:10.1136/bmj.n71. Checklist reproduced/adapted under CC BY 4.0.
