# Supplementary material for: Failure Load and Failure Modes of Indirect Posterior Restorations Combining Occlusal Coverage with Axial Extension: A Systematic Review and Exploratory Meta-Analysis of In Vitro Studies
Source: J Funct Biomater. 2026 Oct 1;17(10):497. doi: 10.3390/jfb17100497 (PMC13705702; doi:10.3390/jfb17100497)
Supplement: Supplementary file 1 [file jfb-17-00497-s001.zip › Table_S1_Search_Strategies.pdf]

Supplementary Materials

Table S1. Complete database-specific search strategies.

All searches covered each database from inception and used no date or language restrictions. Results are the numbers returned before deduplication. No controlled-vocabulary terms were used because the target restoration geometry has no dedicated indexed term. The available documentation supports month-level, but not exact calendar-date, reporting for the two original search rounds. Consistent with the retrospective OSF record, the initial searches were conducted during May–June 2026 and the broadened searches in June 2026. No title/abstract screening, full-text assessment, or inspection of outcome data occurred between the two rounds. Results from both rounds were imported into the same Rayyan review and deduplicated together before screening. Backward and forward citation searching began after final database deduplication; the dates on which the two additional reports were identified and the total number of cited or citing records inspected were not separately logged. All four databases were checked again on 22 July 2026. No record new to the review set was identified or added to screening, so the PRISMA counts were unchanged; the gross number of results displayed during the final check was not separately logged. Thus, 22 July 2026 is the last date on which the databases were consulted, as reported under PRISMA 2020 item 6.

Search round 1 — initial strategy (71 records)

<table>
<tr><th>Database: PubMed | Platform/interface: PubMed, U.S. National Library of Medicine | Records retrieved: 16</th></tr>
<tr><th>Exact search strategy</th></tr>
<tr><td>(vonlay*[tiab] OR veneerlay*[tiab] OR "v onlay"[tiab:~0] OR "veneer onlay"[tiab:~1] OR "buccal occlusal veneer"[tiab:~1] OR "facial occlusal veneer"[tiab:~1] OR "vestibular occlusal veneer"[tiab:~1] OR "palatal occlusal veneer"[tiab:~1] OR "lingual occlusal veneer"[tiab:~1] OR "buccal occlusal onlay"[tiab:~1] OR "facial occlusal onlay"[tiab:~1] OR "vestibular occlusal onlay"[tiab:~1] OR "palatal occlusal onlay"[tiab:~1] OR "lingual occlusal onlay"[tiab:~1] OR "posterior full veneer"[tiab:~1] OR "minimally invasive full veneer"[tiab:~1] OR ((onlay*[tiab] OR veneer*[tiab]) AND (buccal[tiab] OR facial[tiab] OR vestibular[tiab] OR labial[tiab] OR palatal[tiab] OR lingual[tiab]) AND (extension*[tiab] OR coverage[tiab] OR covering[tiab] OR extended[tiab]))) AND (premolar*[tiab] OR molar*[tiab] OR "posterior tooth"[tiab:~0] OR "posterior teeth"[tiab:~0]) AND ("in vitro"[tiab] OR invitro[tiab] OR laborator*[tiab] OR biomechanic*[tiab] OR mechanical[tiab] OR fracture[tiab] OR fatigue[tiab] OR failure[tiab]) AND ((human[tiab] OR extracted[tiab] OR natural[tiab]) AND (tooth[tiab] OR teeth[tiab] OR premolar*[tiab] OR molar*[tiab]))</td></tr>
</table>

<table>
<tr><th>Database: Scopus | Platform/interface: Scopus, Elsevier | Records retrieved: 20</th></tr>
<tr><th>Exact search strategy</th></tr>
<tr><td>TITLE-ABS-KEY ((vonlay* OR veneerlay* OR "v-onlay*" OR "veneer-lay*" OR "veneer onlay*" OR "veneer-onlay*" OR "onlay veneer*" OR "onlay-veneer*" OR "reverse vonlay*" OR "reversed vonlay*" OR "posterior full veneer*" OR "posterior full-veneer*" OR "minimally invasive full veneer*" OR "minimally invasive full-veneer*" OR "bucco-occlusal veneer*" OR "buccal-occlusal veneer*" OR "occluso-buccal veneer*" OR "occlusobuccal veneer*" OR "facio-occlusal veneer*" OR "occluso-facial veneer*" OR "palato-occlusal veneer*" OR "palatal-occlusal veneer*" OR "occluso-palatal veneer*" OR "occlusopalatal veneer*" OR "linguo-occlusal veneer*" OR "lingual-occlusal veneer*" OR "occluso-lingual veneer*" OR "occlusolingual veneer*" OR ((onlay* OR veneer*) W/5 ((occlus* W/3 (buccal OR facial OR vestibular OR labial OR palatal OR lingual)) OR ((buccal OR facial OR vestibular OR labial OR palatal OR lingual) W/3 (extension* OR coverage OR covering OR extended))))) AND (premolar* OR molar* OR "posterior tooth" OR "posterior teeth") AND ("in vitro" OR invitro OR laborator* OR biomechanic* OR mechanical OR fracture OR fatigue OR failure OR "fracture resistance" OR "fracture strength" OR "failure load" OR "load to failure" OR "cyclic loading" OR thermocycl* OR "thermomechanical loading" OR "mechanical loading" OR "stress distribution" OR strain) AND ((human OR extracted OR natural OR intact OR sound OR "caries free") W/3 (tooth OR teeth OR premolar* OR molar*)))</td></tr>
</table>

**Database:** Embase | **Platform/interface:** Embase.com, Elsevier | **Records retrieved: 18**
**Exact search strategy**
(vonlay*:ti,ab,kw OR veneerlay*:ti,ab,kw OR 'v onlay':ti,ab,kw OR 'v-onlay':ti,ab,kw OR 'veneer lay':ti,ab,kw OR 'veneer-lay':ti,ab,kw OR 'veneer onlay':ti,ab,kw OR 'veneer-onlay':ti,ab,kw OR 'onlay veneer':ti,ab,kw OR 'onlay-veneer':ti,ab,kw OR 'reverse vonlay':ti,ab,kw OR 'reversed vonlay':ti,ab,kw OR 'posterior full veneer':ti,ab,kw OR 'posterior full-veneer':ti,ab,kw OR 'minimally invasive full veneer':ti,ab,kw OR 'minimally invasive full-veneer':ti,ab,kw OR 'bucco-occlusal veneer':ti,ab,kw OR 'buccal-occlusal veneer':ti,ab,kw OR 'occluso-buccal veneer':ti,ab,kw OR 'occlusobuccal veneer':ti,ab,kw OR 'facio-occlusal veneer':ti,ab,kw OR 'facial-occlusal veneer':ti,ab,kw OR 'occluso-facial veneer':ti,ab,kw OR 'vestibular-occlusal veneer':ti,ab,kw OR 'occluso-vestibular veneer':ti,ab,kw OR 'palato-occlusal veneer':ti,ab,kw OR 'palatal-occlusal veneer':ti,ab,kw OR 'occluso-palatal veneer':ti,ab,kw OR 'occlusopalatal veneer':ti,ab,kw OR 'linguo-occlusal veneer':ti,ab,kw OR 'lingual-occlusal veneer':ti,ab,kw OR 'occluso-lingual veneer':ti,ab,kw OR 'occlusolingual veneer':ti,ab,kw OR 'bucco-occlusal onlay':ti,ab,kw OR 'buccal-occlusal onlay':ti,ab,kw OR 'occluso-buccal onlay':ti,ab,kw OR 'palato-occlusal onlay':ti,ab,kw OR 'palatal-occlusal onlay':ti,ab,kw OR 'occluso-palatal onlay':ti,ab,kw OR 'linguo-occlusal onlay':ti,ab,kw OR 'lingual-occlusal onlay':ti,ab,kw OR 'occluso-lingual onlay':ti,ab,kw OR ((onlay*:ti,ab,kw OR veneer*:ti,ab,kw) AND occlus*:ti,ab,kw AND (buccal:ti,ab,kw OR facial:ti,ab,kw OR vestibular:ti,ab,kw OR labial:ti,ab,kw OR palatal:ti,ab,kw OR lingual:ti,ab,kw) AND (extension*:ti,ab,kw OR extended:ti,ab,kw OR coverage:ti,ab,kw OR covering:ti,ab,kw))) AND (premolar*:ti,ab,kw OR molar*:ti,ab,kw OR 'posterior tooth':ti,ab,kw OR 'posterior teeth':ti,ab,kw) AND ('in vitro':ti,ab,kw OR invitro:ti,ab,kw OR laborator*:ti,ab,kw OR biomechanic*:ti,ab,kw OR mechanic*:ti,ab,kw OR fracture:ti,ab,kw OR fatigue:ti,ab,kw OR failure:ti,ab,kw OR 'fracture resistance':ti,ab,kw OR 'fracture strength':ti,ab,kw OR 'failure load':ti,ab,kw OR 'load to failure':ti,ab,kw OR 'cyclic loading':ti,ab,kw OR thermocycl*:ti,ab,kw OR 'thermomechanical loading':ti,ab,kw OR 'mechanical loading':ti,ab,kw OR 'stress distribution':ti,ab,kw OR strain:ti,ab,kw) AND (human:ti,ab,kw OR extracted:ti,ab,kw OR natural:ti,ab,kw OR intact:ti,ab,kw OR sound:ti,ab,kw OR 'caries free':ti,ab,kw) AND (tooth:ti,ab,kw OR teeth:ti,ab,kw OR premolar*:ti,ab,kw OR molar*:ti,ab,kw)

**Database:** Web of Science Core Collection | **Platform/interface:** Web of Science Core Collection, Clarivate | **Records retrieved: 17**
**Exact search strategy**
TS=((vonlay* OR veneerlay* OR "v-onlay*" OR "veneer-lay*" OR "veneer onlay*" OR "veneer-onlay*" OR "onlay veneer*" OR "onlay-veneer*" OR "reverse vonlay*" OR "reversed vonlay*" OR "posterior full veneer*" OR "posterior full-veneer*" OR "minimally invasive full veneer*" OR "minimally invasive full-veneer*" OR "bucco-occlusal veneer*" OR "buccal-occlusal veneer*" OR "occluso-buccal veneer*" OR "occlusobuccal veneer*" OR "facio-occlusal veneer*" OR "occluso-facial veneer*" OR "palato-occlusal veneer*" OR "palatal-occlusal veneer*" OR "occluso-palatal veneer*" OR "occlusopalatal veneer*" OR "linguo-occlusal veneer*" OR "lingual-occlusal veneer*" OR "occluso-lingual veneer*" OR "occlusolingual veneer*" OR ((onlay* OR veneer*) NEAR/5 ((occlus* NEAR/3 (buccal OR facial OR vestibular OR labial OR palatal OR lingual)) OR ((buccal OR facial OR vestibular OR labial OR palatal OR lingual) NEAR/3 (extension* OR coverage OR covering OR extended))))) AND (premolar* OR molar* OR "posterior tooth" OR "posterior teeth") AND ("in vitro" OR invitro OR laborator* OR biomechanic* OR mechanical OR fracture OR fatigue OR failure OR "fracture resistance" OR "fracture strength" OR "failure load" OR "load to failure" OR "cyclic loading" OR thermocycl* OR "thermomechanical loading" OR "mechanical loading" OR "stress distribution" OR strain) AND ((human OR extracted OR natural OR intact OR sound OR "caries free") NEAR/3 (tooth OR teeth OR premolar* OR molar*)))

Search round 2 — broadened morphology-based strategy (458 records)

After the first-round records had been imported into Rayyan, the number of records retrieved was judged unexpectedly low for the inconsistently indexed target morphology. Following team discussion and agreement on the revised terms, the strategy was broadened by removing the mandatory substrate block, expanding the terminology and biomechanical-outcome blocks, and conditioning generic restoration terms on both an axial-direction term and an extension term. The interval between the rounds reflected the time required for this discussion and agreement. No records were screened and no study findings, effect estimates, or outcome data were examined between the two searches; broadening was based solely on the number of records retrieved in the first round.

**Database:** PubMed | **Platform/interface:** PubMed, U.S. National Library of Medicine | **Records retrieved: 92**
**Exact search strategy**
((vonlay*[tiab] OR veneerlay*[tiab] OR adhesthetic*[tiab] OR PIAR[tiab] OR "v onlay"[tiab:~0] OR "veneer onlay"[tiab:~1] OR "onlay veneer"[tiab:~1] OR "veneer overlay"[tiab:~1] OR "overlay veneer"[tiab:~1] OR "full veneer"[tiab:~1] OR "complete veneer"[tiab:~1]) OR ((onlay*[tiab] OR veneer*[tiab] OR overlay*[tiab] OR "partial coverage"[tiab:~1] OR "partial crown"[tiab:~1]) AND (buccal[tiab] OR facial[tiab] OR vestibular[tiab] OR labial[tiab] OR palatal[tiab] OR lingual[tiab]) AND (extension*[tiab] OR extend*[tiab] OR coverage[tiab] OR covering[tiab] OR covered[tiab] OR chamfer*[tiab] OR "axial wall"[tiab:~1]))) AND (premolar*[tiab] OR molar*[tiab] OR bicuspid*[tiab] OR "posterior tooth"[tiab:~0] OR "posterior teeth"[tiab:~0] OR "posterior dentition"[tiab:~1]) AND ("in vitro"[tiab] OR invitro[tiab] OR laborator*[tiab] OR biomechanic*[tiab] OR mechanical[tiab] OR fracture*[tiab] OR fatigue[tiab] OR failure*[tiab] OR survival[tiab] OR Weibull[tiab] OR strain*[tiab] OR microstrain*[tiab] OR deflection[tiab] OR deformation[tiab] OR thermocycl*[tiab] OR thermomechanical[tiab] OR "load bearing"[tiab:~1] OR "load to failure"[tiab:~2] OR "failure load"[tiab:~1] OR "cyclic loading"[tiab:~1] OR "step stress"[tiab:~1] OR "chewing simulator"[tiab:~1] OR "mastication simulator"[tiab:~1])

**Database:** Scopus | **Platform/interface:** Scopus, Elsevier | **Records retrieved: 147**
**Exact search strategy**
TITLE-ABS-KEY (((vonlay* OR veneerlay* OR adhesthetic* OR PIAR OR (veneer* W/1 onlay*) OR (veneer* W/1 overlay*) OR (full* W/1 veneer*) OR (complete* W/1 veneer*)) OR ((onlay* OR veneer* OR overlay* OR (partial* W/1 coverage) OR (partial* W/1 crown*)) AND (buccal OR facial OR vestibular OR labial OR palatal OR lingual) AND (extension* OR extend* OR coverage OR covering OR covered OR chamfer* OR "axial wall"))) AND (premolar* OR molar* OR bicuspid* OR "posterior tooth" OR "posterior teeth" OR "posterior dentition") AND ("in vitro" OR invitro OR laborator* OR biomechanic* OR mechanic* OR fracture* OR fatigue OR failure* OR survival OR weibull OR strain* OR microstrain* OR deflection OR deformation OR thermocycl* OR thermomechanic* OR (load W/1 bearing) OR (load W/2 failure) OR (cyclic W/1 loading) OR (step W/1 stress) OR (chewing W/1 simulat*) OR (mastication W/1 simulat*)))

**Database:** Embase | **Platform/interface:** Embase.com, Elsevier | **Records retrieved: 102**
**Exact search strategy**
(vonlay*:ti,ab,kw OR veneerlay*:ti,ab,kw OR adhesthetic*:ti,ab,kw OR piar:ti,ab,kw OR ((veneer* NEAR/1 onlay*):ti,ab,kw) OR ((veneer* NEAR/1 overlay*):ti,ab,kw) OR ((full* NEAR/1 veneer*):ti,ab,kw) OR ((complete* NEAR/1 veneer*):ti,ab,kw) OR ((onlay*:ti,ab,kw OR veneer*:ti,ab,kw OR overlay*:ti,ab,kw OR ((partial* NEAR/1 coverage):ti,ab,kw) OR ((partial* NEAR/1 crown*):ti,ab,kw)) AND (buccal:ti,ab,kw OR facial:ti,ab,kw OR vestibular:ti,ab,kw OR labial:ti,ab,kw OR palatal:ti,ab,kw OR lingual:ti,ab,kw) AND (extension*:ti,ab,kw OR extend*:ti,ab,kw OR coverage:ti,ab,kw OR covering:ti,ab,kw OR covered:ti,ab,kw OR chamfer*:ti,ab,kw OR 'axial wall':ti,ab,kw))) AND (premolar*:ti,ab,kw OR molar*:ti,ab,kw OR bicuspid*:ti,ab,kw OR 'posterior tooth':ti,ab,kw OR 'posterior teeth':ti,ab,kw OR 'posterior dentition':ti,ab,kw) AND ('in vitro':ti,ab,kw OR invitro:ti,ab,kw OR laborator*:ti,ab,kw OR biomechanic*:ti,ab,kw OR mechanic*:ti,ab,kw OR fracture*:ti,ab,kw OR fatigue:ti,ab,kw OR failure*:ti,ab,kw OR survival:ti,ab,kw OR weibull:ti,ab,kw OR strain*:ti,ab,kw OR microstrain*:ti,ab,kw OR deflection:ti,ab,kw OR deformation:ti,ab,kw OR thermocycl*:ti,ab,kw OR thermomechanic*:ti,ab,kw OR 'load bearing':ti,ab,kw OR 'load to failure':ti,ab,kw OR 'failure load':ti,ab,kw OR 'cyclic loading':ti,ab,kw OR 'step stress':ti,ab,kw OR 'chewing simulator':ti,ab,kw OR 'mastication simulator':ti,ab,kw)

**Database:** Web of Science Core Collection | **Platform/interface:** Web of Science Core Collection, Clarivate | **Records retrieved: 117**
**Exact search strategy**
TS=(((vonlay* OR veneerlay* OR adhesthetic* OR PIAR OR (veneer* NEAR/1 onlay*) OR (veneer* NEAR/1 overlay*) OR (full* NEAR/1

**Database:** Web of Science Core Collection | **Platform/interface:** Web of Science Core Collection, Clarivate | **Records retrieved: 117**
veneer*) OR (complete* NEAR/1 veneer*)) OR ((onlay* OR veneer* OR overlay* OR (partial* NEAR/1 coverage) OR (partial* NEAR/1 crown*)) AND (buccal OR facial OR vestibular OR labial OR palatal OR lingual) AND (extension* OR extend* OR coverage OR covering OR covered OR chamfer* OR "axial wall"))) AND (premolar* OR molar* OR bicuspid* OR "posterior tooth" OR "posterior teeth" OR "posterior dentition") AND ("in vitro" OR invitro OR laborator* OR biomechanic* OR mechanical OR fracture* OR fatigue OR failure* OR survival OR Weibull OR strain* OR microstrain* OR deflection OR deformation OR thermocycl* OR thermomechanical OR "load bearing" OR "load to failure" OR "failure load" OR "cyclic loading" OR "step stress" OR "chewing simulator" OR "mastication simulator"))

Overlap management and chronology. Results from the broadened search were imported into the same Rayyan review and deduplicated against the first-round records before screening. Across both rounds, 529 database records yielded 175 unique records after 354 duplicates were removed. The complete deduplicated set, rather than either round separately, then entered title/abstract screening. Thus, the second strategy was developed by explicitly broadening the first strategy and was not informed by eligibility decisions or study outcomes. Citation searching began only after final database deduplication and yielded two additional unique reports that were not among the 175 database records; their individual discovery dates and the total number of cited or citing records inspected were not separately logged. The 22 July 2026 database check added no record to the review set and did not change the flow counts; its gross displayed yield was not separately logged.

Abbreviations: ab, abstract; kw, keyword; ti, title; TITLE-ABS-KEY, title, abstract and keywords; TS, topic search.
