# Supplementary material for: Failure Load and Failure Modes of Indirect Posterior Restorations Combining Occlusal Coverage with Axial Extension: A Systematic Review and Exploratory Meta-Analysis of In Vitro Studies
Source: J Funct Biomater. 2026 Oct 1;17(10):497. doi: 10.3390/jfb17100497 (PMC13705702; doi:10.3390/jfb17100497)
Supplement: Supplementary file 1 [file jfb-17-00497-s001.zip › Table_S2_Risk_of_Bias.pdf]

Table S2. Detailed risk-of-bias assessments using RoBDEMAT and QUIN

Two reviewers independently assessed each included study and resolved discrepancies by consensus. RoBDEMAT was the primary domain-based instrument; QUIN was used as a supplementary structured assessment.

Interpretation note. RoBDEMAT judgments are presented item by item without a total score, consistent with the developers' recommendation. The control-group item was judged against each experiment's stated aim: an appropriate existing-standard or active comparator can be adequate, and absence of an intact-tooth group was not automatically penalized. QUIN percentages are shown for transparency and are not interpreted as continuous measures of methodological quality. RoBDEMAT and QUIN use different criteria and scoring rules; apparently different judgments across the instruments do not necessarily represent a contradiction. Study funding/support is reported separately and is not treated as a risk-of-bias item.

Table S2A. RoBDEMAT item-level judgments and supporting comments

Domain / item	Study	Judgment	Supporting comment
D1 - Planning and allocation<br>1.1 Control group	Al-Mazzaly 2026	Sufficiently reported	IPS e.max CAD was a clearly specified existing-standard material comparator appropriate to the study's material-comparison aim; an intact-tooth group was not required for that aim.
D1 - Planning and allocation<br>1.1 Control group	Guess 2013	Sufficiently reported	Palatal and occlusal onlays at three thicknesses were clearly specified active design comparators appropriate to the study aim; an intact-tooth group was not required.
D1 - Planning and allocation<br>1.1 Control group	Gierthmuehlen 2022	Insufficiently reported	Active onlay comparators were described, but the report states that these control groups were taken from Guess 2013. Their reuse and one discrepant nominally comparable value compromise independent reconstruction.
D1 - Planning and allocation<br>1.1 Control group	Huang 2020	Sufficiently reported	Occlusal-only and full-coverage crown comparators were clearly defined and were appropriate to the stated comparative aim.
D1 - Planning and allocation<br>1.1 Control group	Nguyen-Thi 2026	Sufficiently reported	Healthy tooth, occlusal overlay, and full-coverage crown comparators were clearly defined.
D1 - Planning and allocation<br>1.1 Control group	Sayed 2022	Sufficiently reported	A full-coverage crown comparator was clearly defined.
D1 - Planning and allocation<br>1.1 Control group	Martins 2023	Sufficiently reported	Healthy-tooth and occlusal-only/table-top comparators were clearly defined.
D1 - Planning and allocation<br>1.1 Control group	Moustafa 2024	Sufficiently reported	A conventional occlusal veneer without axial extension was clearly defined as the comparator.
D1 - Planning and allocation<br>1.2 Specimen randomization	Al-Mazzaly 2026	Insufficiently reported	Specimens were described as randomly assigned, but sequence generation and allocation concealment were not reported.
D1 - Planning and allocation<br>1.2 Specimen randomization	Guess 2013	Not reported	The teeth were divided into nine groups; randomization was not reported.
D1 - Planning and allocation<br>1.2 Specimen randomization	Gierthmuehlen 2022	Insufficiently reported	Specimens were described as randomly divided, without details of sequence generation or concealment.
D1 - Planning and allocation<br>1.2 Specimen randomization	Huang 2020	Insufficiently reported	Specimens were described as randomly divided, without procedural details.
D1 - Planning and allocation<br>1.2 Specimen randomization	Nguyen-Thi 2026	Insufficiently reported	Random assignment/distribution was stated, but the process was not described.
D1 - Planning and allocation<br>1.2 Specimen randomization	Sayed 2022	Not reported	No explicit random-allocation procedure was reported.

Domain / item	Study	Judgment	Supporting comment
D1 - Planning and allocation<br>1.2 Specimen randomization	Martins 2023	Insufficiently reported	Random division was stated, but sequence generation and concealment were not described.
D1 - Planning and allocation<br>1.2 Specimen randomization	Moustafa 2024	Sufficiently reported	Specimens were numbered and randomized by sealed envelope, with allocation performed blinded by a co-author.

Domain / item	Study	Judgment	Supporting comment
D1 - Planning and allocation<br>1.3 Sample-size rationale	Al-Mazzaly 2026	Sufficiently reported	An a priori G*Power calculation reported alpha = 0.05, 80% power, and an expected pooled SD; 13 specimens per group were increased to 14.
D1 - Planning and allocation<br>1.3 Sample-size rationale	Guess 2013	Not reported	No sample-size rationale or power calculation was reported.
D1 - Planning and allocation<br>1.3 Sample-size rationale	Gierthmuehlen 2022	Not reported	No sample-size rationale or power calculation was reported.
D1 - Planning and allocation<br>1.3 Sample-size rationale	Huang 2020	Insufficiently reported	No a priori rationale was reported; a retrospective power estimate was provided, which RoBDEMAT discourages.
D1 - Planning and allocation<br>1.3 Sample-size rationale	Nguyen-Thi 2026	Insufficiently reported	The authors justified n = 10 per group by reference to ISO 4049 and comparable published studies. However, no effect-size or power-based calculation was reported, and the applicability of the cited standard to sample-size planning for whole-tooth fracture testing was limited.
D1 - Planning and allocation<br>1.3 Sample-size rationale	Sayed 2022	Not reported	No sample-size rationale or power calculation was reported.
D1 - Planning and allocation<br>1.3 Sample-size rationale	Martins 2023	Insufficiently reported	A power calculation was reported, but its timing was unclear; its a priori status could not be confirmed.
D1 - Planning and allocation<br>1.3 Sample-size rationale	Moustafa 2024	Sufficiently reported	A sample-size calculation using G*Power version 3.1.9.21 was reported; eight specimens per group were selected to allow for possible dropouts.
D2 - Specimen preparation<br>2.1 Specimen/material standardization	Al-Mazzaly 2026	Sufficiently reported	Tooth dimensions were restricted; silicone indices were used; burs were replaced after five preparations; irradiance was reported, but wavelength and tip distance were not.
D2 - Specimen preparation<br>2.1 Specimen/material standardization	Guess 2013	Sufficiently reported	Silicone guides and a periodontal probe were used; wax/restoration thickness was checked; curing wavelength and irradiance were reported, although quantitative tooth-size standardization was limited.
D2 - Specimen preparation<br>2.1 Specimen/material standardization	Gierthmuehlen 2022	Insufficiently reported	Silicone guides and thickness verification were reported; curing irradiance was given, but wavelength was not.
D2 - Specimen preparation<br>2.1 Specimen/material standardization	Huang 2020	Insufficiently reported	Two silicone indices and scheduled bur replacement were reported; curing irradiance and wavelength were not reported.
D2 - Specimen preparation<br>2.1 Specimen/material standardization	Nguyen-Thi 2026	Insufficiently reported	Stepwise protocols and reprinting of nonconforming restorations were reported; curing irradiance was not reported.

Domain / item	Study	Judgment	Supporting comment
D2 - Specimen preparation 2.1 Specimen/material standardization	Sayed 2022	Insufficiently reported	Tooth dimensions, an index, and the ceramic block were specified; light-activation parameters were incompletely reported.
D2 - Specimen preparation 2.1 Specimen/material standardization	Martins 2023	Insufficiently reported	Teeth were dimensionally standardized and checked under magnification; preparation instruments were specified, but photoactivation parameters were incompletely reported.
D2 - Specimen preparation 2.1 Specimen/material standardization	Moustafa 2024	Sufficiently reported	Worn mandibular molars were standardized; material, immediate dentin sealing, depth-cutting burs, and preparation thicknesses were specified.
D2 - Specimen preparation 2.2 Identical experimental conditions	Al-Mazzaly 2026	Insufficiently reported	Storage and mechanical ageing were consistent, but material-specific surface treatments differed and no thermocycling was performed.
D2 - Specimen preparation 2.2 Identical experimental conditions	Guess 2013	Sufficiently reported	Storage, 1.2 million mechanical cycles, and 5,500 thermal cycles were applied consistently across groups.
D2 - Specimen preparation 2.2 Identical experimental conditions	Gierthmuehlen 2022	Insufficiently reported	Fatigue conditions were consistent for the test groups. The report states that control groups came from the earlier Guess 2013 experiment and were tested in an identical setup; the exact extent of overlap across nominally comparable groups could not be fully reconstructed.
D2 - Specimen preparation 2.2 Identical experimental conditions	Huang 2020	Sufficiently reported	Storage, 90-day water conditioning, and 20,000 mechanical cycles were applied consistently.

Domain / item	Study	Judgment	Supporting comment
D2 - Specimen preparation 2.2 Identical experimental conditions	Nguyen-Thi 2026	Insufficiently reported	Storage was standardized, but no mechanical or thermal ageing was performed.
D2 - Specimen preparation 2.2 Identical experimental conditions	Sayed 2022	Insufficiently reported	Thermocycling was applied consistently, but no mechanical ageing was performed.
D2 - Specimen preparation 2.2 Identical experimental conditions	Martins 2023	Sufficiently reported	Periodontal-ligament simulation and the ageing/no-ageing factorial conditions were applied consistently across preparation groups.
D2 - Specimen preparation 2.2 Identical experimental conditions	Moustafa 2024	Sufficiently reported	Periodontal-ligament simulation, 10,000 thermal cycles, and 150,000 mechanical cycles were applied consistently.
D3 - Outcome assessment 3.1 Standardized testing procedures/outcomes	Al-Mazzaly 2026	Insufficiently reported	An 8-mm steel ball, 0.5 mm/min crosshead speed, and a 0.6-mm rubber sheet were reported; the stated loading direction was ambiguous.
D3 - Outcome assessment 3.1 Standardized testing procedures/outcomes	Guess 2013	Insufficiently reported	A steel wedge at the central fissure, 0.1 mm/min, and a tin sheet were reported, but the linear-contact setup limits comparability with sphere-loading studies.

Domain / item	Study	Judgment	Supporting comment
D3 - Outcome assessment 3.1 Standardized testing procedures/outcomes	Gierthmuehlen 2022	Sufficiently reported	A 6-mm steel sphere, 1.5 mm/min, the fatigue contact point, and microscopic failure assessment were reported.
D3 - Outcome assessment 3.1 Standardized testing procedures/outcomes	Huang 2020	Sufficiently reported	A 4-mm indenter contacting both cusps, 0.5 mm/min, a tin sheet, stereomicroscopy, and SEM fractography were reported.
D3 - Outcome assessment 3.1 Standardized testing procedures/outcomes	Nguyen-Thi 2026	Sufficiently reported	A rounded 4-mm indenter contacting both cusps, 0.5 mm/min, a tin sheet, water-bath testing, and visual/photographic assessment were reported.
D3 - Outcome assessment 3.1 Standardized testing procedures/outcomes	Sayed 2022	Insufficiently reported	The report contains an internal discrepancy in indenter diameter (5.8 versus 4 mm), preventing an unambiguous reconstruction of the test.
D3 - Outcome assessment 3.1 Standardized testing procedures/outcomes	Martins 2023	Insufficiently reported	A universal testing machine and stereomicroscopic failure assessment were reported, but indenter details were incomplete.
D3 - Outcome assessment 3.1 Standardized testing procedures/outcomes	Moustafa 2024	Insufficiently reported	Vertical loading in a universal testing machine was reported, but indenter dimensions and contact geometry were incompletely described.
D3 - Outcome assessment 3.2 Blinding of test operator/outcome assessor	Al-Mazzaly 2026	Not reported	Blinding was not reported.
D3 - Outcome assessment 3.2 Blinding of test operator/outcome assessor	Guess 2013	Not reported	Blinding was not reported.
D3 - Outcome assessment 3.2 Blinding of test operator/outcome assessor	Gierthmuehlen 2022	Not reported	Blinding was not reported.
D3 - Outcome assessment 3.2 Blinding of test operator/outcome assessor	Huang 2020	Not reported	Blinding was not reported.
D3 - Outcome assessment 3.2 Blinding of test operator/outcome assessor	Nguyen-Thi 2026	Not reported	Blinding was not reported.

Domain / item	Study	Judgment	Supporting comment
D3 - Outcome assessment<br>3.2 Blinding of test operator/outcome assessor	Sayed 2022	Not reported	Blinding was not reported.
D3 - Outcome assessment<br>3.2 Blinding of test operator/outcome assessor	Martins 2023	Not reported	Blinding was not reported.
D3 - Outcome assessment<br>3.2 Blinding of test operator/outcome assessor	Moustafa 2024	Not reported	Allocation was blinded, but blinding of the test operator or outcome assessor was not reported.
D4 - Data treatment and reporting<br>4.1 Statistical analysis	Al-Mazzaly 2026	Sufficiently reported	Software, distribution checks, one-way ANOVA, Tukey testing, chi-square/Fisher testing, and alpha were reported.
D4 - Data treatment and reporting<br>4.1 Statistical analysis	Guess 2013	Sufficiently reported	Software, two-way ANOVA with interaction, Tukey-Kramer testing, residual diagnostics, and influence assessment were reported.
D4 - Data treatment and reporting<br>4.1 Statistical analysis	Gierthmuehlen 2022	Sufficiently reported	Software, linear regression, multiple-comparison testing, paired testing, and dedicated statistical support were reported.
D4 - Data treatment and reporting<br>4.1 Statistical analysis	Huang 2020	Sufficiently reported	Software, normality and variance checks, ANOVA, Tukey, and Fisher exact testing were reported.
D4 - Data treatment and reporting<br>4.1 Statistical analysis	Nguyen-Thi 2026	Sufficiently reported	Software, one-way ANOVA, Tukey, Fisher exact testing, and alpha were reported.
D4 - Data treatment and reporting<br>4.1 Statistical analysis	Sayed 2022	Insufficiently reported	One-way ANOVA and LSD post hoc testing were reported, but statistical software and distribution checks were not.
D4 - Data treatment and reporting<br>4.1 Statistical analysis	Martins 2023	Sufficiently reported	A generalized linear model was used after ANOVA assumptions were not met; preparation, cycling, and interaction effects were reported.
D4 - Data treatment and reporting<br>4.1 Statistical analysis	Moustafa 2024	Insufficiently reported	A between-group P value was reported, but the statistical test and software were not described clearly.
D4 - Data treatment and reporting<br>4.2 Outcome reporting	Al-Mazzaly 2026	Sufficiently reported	Mean, SD, median, range, pairwise comparisons with 95% CIs, and complete failure-mode data were reported.
D4 - Data treatment and reporting<br>4.2 Outcome reporting	Guess 2013	Insufficiently reported	Mean, SD, median, quartiles, range, and failure modes were reported; confidence intervals were absent.
D4 - Data treatment and reporting<br>4.2 Outcome reporting	Gierthmuehlen 2022	Insufficiently reported	Descriptive and failure-mode data were reported. Two corresponding group means were identical to Guess 2013, whereas a third nominally comparable mean differed between reports, complicating complete reconstruction of the reused control data.
D4 - Data treatment and reporting<br>4.2 Outcome reporting	Huang 2020	Insufficiently reported	Mean, SD, failure modes, and SEM fractography were reported; confidence intervals and quartiles were absent.
D4 - Data treatment and reporting<br>4.2 Outcome reporting	Nguyen-Thi 2026	Insufficiently reported	Mean, SD, range, failure modes, and preparation time were reported; confidence intervals were absent.
D4 - Data treatment and reporting<br>4.2 Outcome reporting	Sayed 2022	Insufficiently reported	Mean, SD, range, and failure modes were reported, but failure-mode percentages summed to 96%, with an unexplained 4% post hoc exclusion and no group-specific breakdown.
D4 - Data treatment and reporting<br>4.2 Outcome reporting	Martins 2023	Insufficiently reported	Mean and SD were reported by preparation and cycling condition with a failure-mode figure; confidence intervals were absent.

Domain / item	Study	Judgment	Supporting comment
D4 - Data treatment and reporting 4.2 Outcome reporting	Moustafa 2024	Insufficiently reported	Means, SDs, medians/ranges, and a fracture-mode histogram were reported; confidence intervals were absent, and the fracture-mode categories accounted for seven of eight specimens per group.

RoBDEMAT reporting/judgment labels used in this review: sufficiently reported; insufficiently reported; not reported; not applicable. No item was judged not applicable. Control adequacy was assessed relative to the stated study aim, not by requiring an intact-tooth group for every experiment.

Table S2B. QUIN item-level scores

Scoring: 2 = adequately specified; 1 = inadequately specified; 0 = not specified; NA = not applicable. Final percentage = (total score x 100) / (2 x number of applicable criteria). Categories: >70%, low risk; 50%-70%, medium risk; <50%, high risk.

QUIN risk categories reflect the domains included in that instrument and should not be interpreted as an overall measure of experimental or clinical applicability. For example, Nguyen-Thi 2026 was categorized as low risk by QUIN despite the absence of mechanical or thermal ageing.

No.	Criterion (paraphrased)	Al-Mazzaly 2026	Guess 2013	Gierthmuehlen 2022	Huang 2020	Nguyen-Thi 2026	Sayed 2022	Martins 2023	Moustafa 2024
1	Clearly stated aims/objectives	2	2	2	2	2	1	2	2
2	Detailed sample-size calculation	2	0	0	1	1	0	2	2
3	Detailed sampling technique	2	2	2	2	2	2	2	2
4	Details of the comparison group	0	1	1	2	2	1	2	1
5	Detailed methodology and standardization	2	2	2	2	2	1	2	1
6	Operator details, training, and calibration	1	1	1	1	1	1	1	1
7	Randomization procedure	1	0	1	1	1	0	1	2
8	Outcome-measurement method	1	1	1	2	2	0	1	1
9	Outcome-assessor details, training, and calibration	0	0	0	0	0	0	0	0
10	Blinding	0	0	0	0	0	0	0	1
11	Statistical analysis	2	2	2	2	2	1	2	1
12	Outcome presentation	2	1	0	1	2	0	1	1

Table S2C. QUIN summary and principal interpretive concerns

Study	Total score	Final score	QUIN category	Principal RoBDEMAT / interpretive notes
Nguyen-Thi 2026	17/24	70.8%	Low risk	No periodontal-ligament simulation; no assessor blinding; no mechanical ageing.
Huang 2020	16/24	66.7%	Medium risk	Small groups; no assessor blinding; the same comparator contributed to three eligible-arm contrasts before arm combination.
Martins 2023	16/24	66.7%	Medium risk	Only semi-direct study; non-axial loading geometry; timing of the power calculation was unclear.
Al-Mazzaly 2026	15/24	62.5%	Medium risk	A priori sample-size calculation; no thermocycling; material comparison without an occlusal-only design control.

Study	Total score	Final score	QUIN category	Principal RoBDEMAT / interpretive notes
Moustafa 2024	15/24	62.5%	Medium risk	Sample-size calculation reported; indenter geometry incompletely reported; fracture-mode categories accounted for seven of eight specimens per group.
Guess 2013	12/24	50.0%	Medium risk	Randomization and assessor blinding not reported; industry support reported (contextual only; not coded as methodological bias); control data were reported again in a later study.
Gierthmuehlen 2022	12/24	50.0%	Medium risk	Control groups reported as originating from Guess 2013; one nominally comparable descriptive value differed between reports; industry support reported (contextual only; not coded as methodological bias).
Sayed 2022	7/24	29.2%	High risk	Internal reporting inconsistencies; group-specific failure modes unavailable; discrepant indenter diameter.

References

1. Delgado AHS, et al. RoBDEMAT: A risk of bias tool and guideline to support reporting of pre-clinical dental materials research and assessment of systematic reviews. J Dent. 2022;127:104350. doi:10.1016/j.jdent.2022.104350.
2. Sheth VH, et al. Development and validation of a risk-of-bias tool for assessing in vitro studies conducted in dentistry: The QUIN. J Prosthet Dent. 2024;131:1038-1042. doi:10.1016/j.prosdent.2022.05.019.
