# Supplementary material for: Failure Load and Failure Modes of Indirect Posterior Restorations Combining Occlusal Coverage with Axial Extension: A Systematic Review and Exploratory Meta-Analysis of In Vitro Studies
Source: J Funct Biomater. 2026 Oct 1;17(10):497. doi: 10.3390/jfb17100497 (PMC13705702; doi:10.3390/jfb17100497)
Supplement: Supplementary file 1 [file jfb-17-00497-s001.zip › Table_S3_Full_Text_Exclusions.pdf]

Supplementary Table S3. Reports excluded after full-text assessment and primary reasons for exclusion

One mutually exclusive primary reason for exclusion is reported for each full-text report. Reasons were assigned at report level against the morphology-, specimen-, and design-based eligibility criteria; no exclusion was based on effect direction or outcome magnitude. Bibliographic citations satisfy PRISMA 2020 item 16b.

No.	Title	Primary reason for exclusion	Bibliographic citation
1	Fatigue behavior of endodontically treated maxillary premolars with MOD defects under different minimally invasive restorations.	Ineligible preparation morphology – incomplete occlusal coverage	Chen Y, Chen D, Ding H, et al. Clin Oral Investig. 2022;26(1):197–206. doi:10.1007/s00784-021-03991-9.
2	Monolithic Zirconia Partial Coverage Restorations: An In Vitro Mastication Simulation Study.	Ineligible preparation morphology – incomplete occlusal coverage	Gupta S, Abdulmajeed A, Donovan T, et al. J Prosthodont. 2021;30(1):76–82. doi:10.1111/jopr.13287.
3	Fracture strength of non-invasively reinforced MOD cavities on endodontically treated teeth.	Ineligible preparation morphology – incomplete occlusal coverage	Daher R, Ardu S, Di Bella E, et al. Odontology. 2021;109(2):368–375. doi:10.1007/s10266-020-00552-6.
4	Load capacity of occlusal veneers of different restorative CAD/CAM materials under lateral static loading.	Ineligible preparation morphology – no veneer-like axial extension	Zamzam H, Olivares A, Fok A. J Mech Behav Biomed Mater. 2021;115:104290. doi:10.1016/j.jmbbm.2020.104290.
5	Evaluation of Marginal Integrity of Lithium Disilicate Vonlays versus Celtra Duo Vonlays Restoring Premolars (In Vitro Study).	Artificial teeth or replicas	El-Naggar HAE, El-Khodary N, Hashem AB, Khairallah L. Adv Dent J. 2023;5(2):276–285. doi:10.21608/adjc.2023.171938.1194.
6	Influence of cavity preparation design on fracture resistance of posterior leucite-reinforced ceramic restorations.	Ineligible preparation morphology – incomplete occlusal coverage	Soares CJ, Martins LRM, Fonseca RB, Correr-Sobrinho L, Fernandes Neto AJ. J Prosthet Dent. 2006;95(6):421–429. doi:10.1016/j.prosdent.2006.03.022.
7	Fracture strength and failure patterns of different restoration approaches in endodontically treated maxillary premolars: an in vitro study with fractographic analysis.	Ineligible preparation morphology – complete removal of cuspal anatomy	Amin AY, Hamdy A, Wahsh M, et al. BMC Oral Health. 2026;26:634. doi:10.1186/s12903-026-08064-5.
8	Fatigue Performance and Failure Loads of Composition-Gradient Multilayer Zirconia: Partial-Coverage Restorations Versus Single Crowns at Different Thicknesses.	Artificial teeth or replicas	Spitznagel FA, Marksteiner A, Prott LS, et al. J Esthet Restor Dent. 2026;38(3):539–552. doi:10.1111/jerd.70066.
9	Effect of preparation designs and CAD-CAM materials on step-stress fatigue survival of premolar partial coverage restorations: An in-vitro study with fractographic analysis.	Artificial teeth or replicas	Nasr DM, Abdelraheem IM, Watts DC, et al. Dent Mater. 2025;41(2):122–133. doi:10.1016/j.dental.2024.11.003.
10	Fracture resistance and fractographic analysis of pressable glass-ceramics with different partial coverage designs for maxillary premolars.	Artificial teeth or replicas	Abdelaal AM, Kehela HA, Holiel AA. BMC Oral Health. 2024;24:1078. doi:10.1186/s12903-024-04809-2.
11	Minimally invasive CAD/CAM lithium disilicate partial-coverage restorations show superior in-vitro fatigue performance than single crowns.	Artificial teeth or replicas	Spitznagel FA, Prott LS, Hoppe JS, et al. J Esthet Restor Dent. 2024;36(1):94–106. doi:10.1111/jerd.13169.
12	Impact of different axial wall designs on the fracture strength and stress distribution of ceramic restorations in mandibular first molar.	Ineligible preparation morphology – no veneer-like axial extension	Luo B, Sun X, He L, et al. BMC Oral Health. 2022;22:549. doi:10.1186/s12903-022-02577-5.
13	Fracture resistance of bonded ceramic overlay restorations prepared in various designs.	Ineligible preparation morphology – no veneer-like axial extension	Channarong W, Lohawiboonkij N, Jaleyasuthumkul P, et al. Sci Rep. 2022;12:16599. doi:10.1038/s41598-022-21167-7.
14	Comparison of posterior indirect adhesive restorations (PIAR) with different preparation designs according to the adhesthetics classification. Part 1: Effects on the fracture resistance.	Ineligible preparation morphology – no veneer-like axial extension	Ferraris F, Sammarco E, Romano G, Cincera S, Marchesi G. Int J Esthet Dent. 2021;16(2):144–167. PMID:33969972.
15	Impact of Various Cavity-Preparation Designs on Fracture Resistance and Failure Mode of CAD/CAM Fabricated Ceramic Inlays and Onlays.	Ineligible preparation morphology – incomplete occlusal coverage	Elkaffas AA, Alshehri AM, Alqahtani AR, Albaijan RS, Soliman TA. Appl Sci. 2024;14(9):3816. doi:10.3390/app14093816.
16	Timing and method of restoration affect fracture resistance of endodontically treated teeth: An in vitro study.	Ineligible preparation morphology – incomplete occlusal coverage	Yuan Y, Chen P. Medicine (Baltimore). 2025;104(51):e46421. doi:10.1097/MD.0000000000046421.
17	Comparative evaluation of the marginal fitness of vonlay nanoceramic hybrid restorations using different computer-aided imaging techniques: an in-vitro study.	Artificial teeth or replicas	Salem ZM, Shakal M, Eldessouky R. Tanta Dent J. 2023;20:20–26. doi:10.4103/tdj.tdj_48_22.

No.	Title	Primary reason for exclusion	Bibliographic citation
18	Stress Distribution in Modified Veneer Crowns: 3D Finite Element Analysis.	Finite-element analysis without experimental testing	Madruga CFL, Ramos GF, Borges ALS, et al. Oral. 2021;1(3):272–280. doi:10.3390/oral1030026.
19	Fracture resistance of maxillary premolar veneers using various preparation designs.	Artificial teeth or replicas	Hernandez A, Jurado CA, Antal MA, et al. J Prosthodont. 2026. doi:10.1111/jopr.70176.
20	Tooth-cusp preservation with lithium disilicate onlay restorations: A fatigue resistance study.	Ineligible preparation morphology – incomplete occlusal coverage	Griffis E, Abd Alraheam I, Boushell L, Donovan T, Fasbinder D, Sulaiman TA. J Esthet Restor Dent. 2022;34(3):512–518. doi:10.1111/jerd.12666.

Summary of primary exclusion categories: ineligible preparation/restoration morphology, n = 12; artificial teeth or replicas, n = 7; finite-element analysis without experimental testing, n = 1.
