# Supplementary material for: Failure Load and Failure Modes of Indirect Posterior Restorations Combining Occlusal Coverage with Axial Extension: A Systematic Review and Exploratory Meta-Analysis of In Vitro Studies
Source: J Funct Biomater. 2026 Oct 1;17(10):497. doi: 10.3390/jfb17100497 (PMC13705702; doi:10.3390/jfb17100497)
Supplement: Supplementary file 1 [file jfb-17-00497-s001.zip › Table_S4_Study_Level_Outcomes.pdf]

Supplementary Table S4. Study-level outcome data contributing to the quantitative syntheses

Table S4A. Primary quantitative failure-load comparison: single-surface axial extension versus occlusal-only

Study	Comparator	Extension arm(s)	AE n; mean ± SD (N)	Comparator n; mean ± SD (N)	MD (N)	95% CI (N)
Huang 2020	Occlusal-only	OF	8; 1999.0 ± 432.4	8; 2153.5 ± 405.7	-154.6	-565.4 to 256.3
Nguyen-Thi 2026	Occlusal-only	VL	10; 1667.4 ± 497.5	10; 1842.8 ± 560.5	-175.5	-640.0 to 289.0
Martins 2023	Occlusal-only	OV; cycling strata combined	20; 2119.6 ± 695.1	20; 2208.3 ± 726.0	-88.8	-529.3 to 351.7
Guess 2013	Occlusal-only	Complete veneer; 3 thicknesses	48; 1093.7 ± 365.8	48; 1022.7 ± 357.9	+71.0	-73.8 to 215.8
Moustafa 2024	Occlusal-only	Buccal + lingual single-surface	15; 2558.5 ± 670.9	7; 2909.4 ± 905.8	-350.9	-1102.9 to 401.1

Notes: MD = axial-extension minus comparator. Only one-surface extension designs were retained, with independent thickness or cycling strata combined within study and shared comparators counted once. This restriction was finalized post hoc after peer review. Guess uses 833 N from the detailed table. Using the discrepant 883 N abstract value produced a primary MD of −23 N (95% CI −209 to 163) and a broad-analysis MD of −141 N (95% CI −429 to 146); the interpretation was unchanged. The corresponding analyses are reproducible in File S1.

Table S4B. Broad all-eligible-geometry sensitivity and unpooled crown comparisons

Role	Study	Comparator	AE n; mean ± SD (N)	Comparator n; mean ± SD (N)	MD (N)	95% CI (N)
Broad sensitivity	Huang 2020	Occlusal-only	24; 1711.9 ± 392.7	8; 2153.5 ± 405.7	-441.7	-763.7 to -119.6
Broad sensitivity	Nguyen-Thi 2026	Occlusal-only	10; 1667.4 ± 497.5	10; 1842.8 ± 560.5	-175.5	-640.0 to 289.0
Broad sensitivity	Martins 2023	Occlusal-only	40; 2129.7 ± 706.8	20; 2208.3 ± 726.0	-78.6	-464.9 to 307.7
Broad sensitivity	Guess 2013	Occlusal-only	48; 1093.7 ± 365.8	48; 1022.7 ± 357.9	+71.0	-73.8 to 215.8
Broad sensitivity	Moustafa 2024	Occlusal-only	15; 2558.5 ± 670.9	7; 2909.4 ± 905.8	-350.9	-1102.9 to 401.1
Crown; unpooled	Huang 2020	Full crown	24; 1711.9 ± 392.7	8; 1428.4 ± 172.4	+283.4	86.1 to 480.8
Crown; unpooled	Nguyen-Thi 2026	Full crown	10; 1667.4 ± 497.5	10; 1236.8 ± 497.6	+430.6	-5.6 to 866.7
Crown; unpooled	Sayed 2022	Full crown	10; 745.5 ± 96.5	10; 1015.7 ± 38.9	-270.2	-334.7 to -205.7

Notes: The broad sensitivity includes every eligible axial-extension arm and therefore mixes single- and multi-surface geometries. Crown effects are shown study by study only; no crown summary was calculated because the three comparisons were directionally discordant and mechanically incompatible.

Table S4C. Review-defined severe tooth/root-involving failure

Study	Comparator	Axial-extension events/total	Comparator events/total	Risk ratio	95% CI	Continuity correction
Huang 2020	Occlusal-only	6/24	1/8	2.00	0.28 to 14.20	None
Nguyen-Thi 2026	Occlusal-only	2/10	0/10	5.00	0.27 to 92.62	0.5
Martins 2023	Occlusal-only	16/40	7/20	1.14	0.56 to 2.32	None

Study	Comparator	Axial-extension events/total	Comparator events/total	Risk ratio	95% CI	Continuity correction
Guess 2013	Occlusal-only	12/48	14/48	0.86	0.44 to 1.66	None
Moustafa 2024	Occlusal-only	3/14	0/7	3.73	0.22 to 63.66	0.5
Huang 2020	Full-coverage crown	6/24	0/8	4.68	0.29 to 74.96	0.5
Nguyen-Thi 2026	Full-coverage crown	2/10	6/10	0.33	0.09 to 1.27	None

Notes: Exact events and totals are the primary presentation. RR < 1 favours axial extension. For review-defined severe failure, all eligible axial-extension arms with group-resolved events were combined within study; this synthesis therefore averages single- and multi-surface geometries and is treated as exploratory. The harmonized review-defined category requires an explicit fracture below the cementoenamel junction or involving the root and is not equivalent to a prospectively defined clinical judgment of non-restorability. A 0.5 correction was applied only to zero-cell studies for individual and exploratory random-effects RRs. Crown comparisons were not pooled.

Table S4D. Model-level quantitative summary

Analysis	Effect	Model	Estimate	95% CI	95% prediction interval	τ^2	I-squared
Single-surface	RoM	REML + Hartung-Knapp	0.98	0.88 to 1.09	0.87 to 1.10	0.00046 (log scale)	4.1%
Single-surface	MD (N)	REML + Hartung-Knapp	-23.1	-194.7 to 148.5	-294.3 to 248.1	5,718.4 N-squared	13.7%
Broad sensitivity	RoM	REML + Hartung-Knapp	0.93	0.79 to 1.08	0.68 to 1.26	0.00927 (log scale)	50.6%
Broad sensitivity	MD (N)	REML + Hartung-Knapp	-145.0	-424.7 to 134.7	-733.8 to 443.8	34,825.8 N-squared	54.5%
All eligible geometries; severe failure (exploratory)	RR	REML + Hartung-Knapp	1.10	0.65 to 1.86	Not emphasized	0	0.0%
Severe failure; sensitivity	RR	Mantel-Haenszel equal-effects	1.19	0.75 to 1.90	NA	NA	0.0%

Notes: The structured narrative synthesis remains primary. The single-surface model is the principal quantitative failure-load comparison but was finalized after peer review and is explicitly post hoc. The severe-failure model combines all eligible geometries with group-resolved events and is exploratory. Prediction intervals are descriptive with only five studies. All confidence intervals shown in Table S4D include the null; none establishes equivalence, non-inferiority, benefit, or absence of harm. I-squared values are reported for completeness and should be interpreted cautiously with so few studies. In Guess, a common MOD inlay preparation, including the same isthmus and proximal boxes, was present in both axial-extension and occlusal-only arms; the complete-veneer arms added a labial extension.

Table S4E. Random-effects model weights (%)

Study	Single MD	Single RoM	Broad MD	Broad RoM	Severe RR
Huang 2020	13.4	19.6	22.8	23.9	5.4
Nguyen-Thi 2026	10.8	11.3	15.5	13.8	2.4
Martins 2023	11.8	18.7	19.1	21.8	41.6
Guess 2013	59.6	39.3	34.9	26.8	47.9
Moustafa 2024	4.4	11.1	7.7	13.7	2.6

Notes: Weights are normalized within each fitted model. MD, mean difference; RoM, ratio of means; RR, risk ratio.

Abbreviations: AE, axial-extension; CI, confidence interval; MD, mean difference; NA, not applicable; RoM, ratio of means; RR, risk ratio; SD, standard deviation.
